# Author Correction: SVSBI: sequence-based virtual screening of biomolecular interactions

**DOI:** 10.1038/s42003-024-06554-2

**Published:** 2024-07-15

**Authors:** Li Shen, Hongsong Feng, Yuchi Qiu, Guo-Wei Wei

**Affiliations:** 1 Department of Mathematics, Michigan State University, East Lansing, MI 48824 USA; 2 Department of Electrical and Computer Engineering, Michigan State University, East Lansing, MI 48824 USA; 3 Department of Biochemistry and Molecular Biology, Michigan State University, East Lansing, MI 48824 USA

**Keywords:** Machine learning, Computational models

Correction to: *Communications Biology* 10.1038/s42003-023-04866-3, published online 18 May 2023

The original version of the article contained error in the text which should be 1695 instead of 1795 in the below mentioned two sentences in Results and Methods sections.

“We collect and curate a set of 1695 PPI complexes (Datasets) in the PDBbind database” and “We selected 1695 samples with only two different sub-chain sequences”

In addition, the revised version of supplementary file replaced with the original version. The original article has been corrected.

