# Supplementary material for: SVSBI: sequence-based virtual screening of biomolecular interactions
Source: Commun Biol. 2023 May 18;6:536. doi: 10.1038/s42003-023-04866-3 (PMC10195826; doi:10.1038/s42003-023-04866-3)
Supplement: Supplementary file 2 — Supplementary Information [file 42003_2023_4866_MOESM2_ESM.pdf]

Supplementary Information for SVSBI: Sequence-based virtual screening of biomolecular interactions

Li Shen[1], Hongsong Feng[1] , Yuchi Qiu[1] and Guo-Wei Wei[1,2,3*]
[1] Department of Mathematics,
Michigan State University, East Lansing, MI 48824, USA.
[2] Department of Electrical and Computer Engineering,
Michigan State University, East Lansing, MI 48824, USA.
[3] Department of Biochemistry and Molecular Biology,
Michigan State University, East Lansing, MI 48824, USA.

June 25, 2024

*Corresponding author. E-mail: weig@msu.edu

Supplementary Note 1 Additional evaluation metrics

In this study, we used, accuracy (ACC), precision (Pre), sensitivity (Se), Matthews correlation coefficient (MCC), and F1-score to evaluate the performance of our models for classification problems. These evaluation metrics are defined as below:

$$ACC = \frac{TP + TN}{TP + TN + FP + FN} \quad \text{(Supplement Equation 1)}$$

$$Pre = \frac{TP}{TP + FP} \quad \text{(Supplement Equation 2)}$$

$$Se = \frac{TP}{TP + FN} \quad \text{(Supplement Equation 3)}$$

$$MCC = \frac{TP \times TN - FP \times FN}{\sqrt{(TP + FP)(TP + FN)(TN + FP)(TN + FN)}} \quad \text{(Supplement Equation 4)}$$

$$\text{F1-score} = \frac{2 \times Pre \times Se}{Pre + Se}, \quad \text{(Supplement Equation 5)}$$

where TP (True positive) indicates the number of positive samples that are predicted as positive; false negative (FN_i) indicates the number of negative samples that are predicted as negative; true negative (TN_i) indicates the number of negative samples that are predicted as negative; and false positive (FP_i) indicates the number of negative samples that are predicted as negative.

We also used receiver operating characteristic (ROC) curve [1] and Area under ROC curve (AUC) to evaluate of our model at all decision thresholds. An ROC curve plots True-positive Rate (TPR) vs. False-positive Rate at different decision thresholds. Lowering the decision threshold will classifies more instances as positive, thus increasing both TPR and FPR. AUC measures the entire two-dimensional area underneath the ROC curve, ranging from 0 to 1. The higher the AUC, the better the performance of the model at distinguishing the positive and negative classes.

Furthermore, the Pearson correlation coefficient (R_p) is used to evaluate our models in regression tasks, and it is defined as follows:

$$R_\text{p} = \frac{\sum(x_i - \bar{x})(y_i - \bar{y})}{\sqrt{\sum(x_i - \bar{x})^2(y_i - \bar{y})^2}}, \quad \text{(Supplement Equation 6)}$$

where x_i is the value of the x variable in the i-th sample, y_i is the value of y variable in the i-th sample, $\bar{x}$ is mean value of the x variable and $\bar{y}$ is mean value of the y variable. Also, the root mean squared error (RMSE) is applied,and it is defined as below,

$$\text{RMSE} = \sqrt{\frac{1}{n}\sum_{i=1}^{n}(y_i - \hat{y_i})^2}, \quad \text{(Supplement Equation 7)}$$

where y_i and $\hat{y_i}$ are predicted and true value of the i-th sample, respectively.

Supplementary Note 2 Additional results

Dataset	Method	Accuracy	Precision	Sensitivity	F1-score	MCC
H. sapiens	SVM-NVDT [2]	0.9541	0.9159	**1.0000**	0.9561	0.9121
	RF-NVDT [2]	0.9541	0.9159	**1.0000**	0.9541	0.9121
	**SVS**	**0.9959±0.0045**	**1.0000±0.0000**	0.9918±0.0045	**0.9959±0.0023**	**0.9918**
M. musculus	SVM-NVDT [2]	0.9643	0.9310	0.8971	0.9474	0.8971
	RF-NVDT [2]	0.9483	**1.0000**	0.8966	0.9014	0.9455
	**SVS**	**0.9928±0.0072**	0.9942±0.0907	**0.9914±0.0068**	**0.9928±0.0503**	**0.9856**
S. cerevisiae	SVM-AC [3]	0.8735	0.8782	0.8730	0.8734	0.7509
	kNN-CTD [4]	0.8615	0.9024	0.8103	0.8539	NA
	EELM-PCA [5]	0.8699	0.8759	0.8615	0.8686	0.7736
	SVM-MCD [6]	0.9136	0.9194	0.9067	0.9130	0.8421
	RF-PR-LPQ [7]	0.9392	0.9645	0.9110	0.9380	0.8856
	MLP [8]	0.9443	0.9665	0.9206	0.9430	0.8897
	PCVMZM [9]	0.9448	0.9392	0.9513	NA	0.8959
	DPPI [10]	0.9455	0.9668	0.9224	0.9441	NA
	LightGBM [11]	0.9507	0.9782	0.9221	NA	0.9030
	PIPR [12]	0.9709	0.9700	0.9717	0.9709	0.9417
	StackPPI [13]	0.9464	0.9633	0.9281	NA	0.8934
	TAGPPI [14]	0.9781	0.9810	0.9826	0.9780	0.9563
	SVM-NVDT [2]	0.9920	0.9935	0.9903	NA	0.9839
	**SVS**	**0.9964±0.0038**	**0.9998±0.0375**	**0.9998±0.0039**	**0.9964±0.0195**	**0.9929**
H. pylori	HKNN [15]	0.8400	0.8400	0.8600	NA	NA
	DCT+SMR [16]	0.8674	0.8701	0.8643	NA	0.7699
	S-products [17]	0.8340	0.8570	0.7990	NA	NA
	P-bootstrap [18]	0.7580	0.6980	0.8020	NA	NA
	SVM-MCD [6]	0.8491	0.8612	0.8324	NA	0.7440
	LightGBM [11]	0.8779	0.8787	0.8772	NA	0.7561
	XGBoost [13]	0.8927	0.9037	0.8793	NA	0.7859
	PCVMZM [9]	0.9125	0.9006	0.9205	NA	0.8404
	DeepPPI [8]	0.8623	0.8432	0.8944	NA	0.7263
	StackPPI [13]	0.8927	0.9037	0.8793	NA	0.7859
	SVM-NVDT [2]	0.9856	0.9836	0.9877	NA	0.9712
	**SVS**	**0.9993±0.0014**	**1.0000±0.0000**	**0.9986±0.0019**	**0.9993±0.0007**	**0.9986**
D. melanogaster	NVD [19]	0.7978	0.8487	0.7247	0.7818	0.6020
	RFDC [20]	0.8904	0.8971	0.8820	0.8895	0.7810
	SVM-NVDT [2]	0.9494	0.9762	0.9213	0.9480	0.9003
	**SVS**	**0.9869±0.0164**	**1.0000±0.0000**	**0.9738±0.0164**	**0.9867±0.0084**	**0.9742**

Note: NA means not available. Standard deviation of MCC is not provided as it is ill-defined for many folds of our results.

Supplementary Table 1: Comparison of our method with previous models for protein-protein interaction identification. The best scores of each dataset are marked in bold.

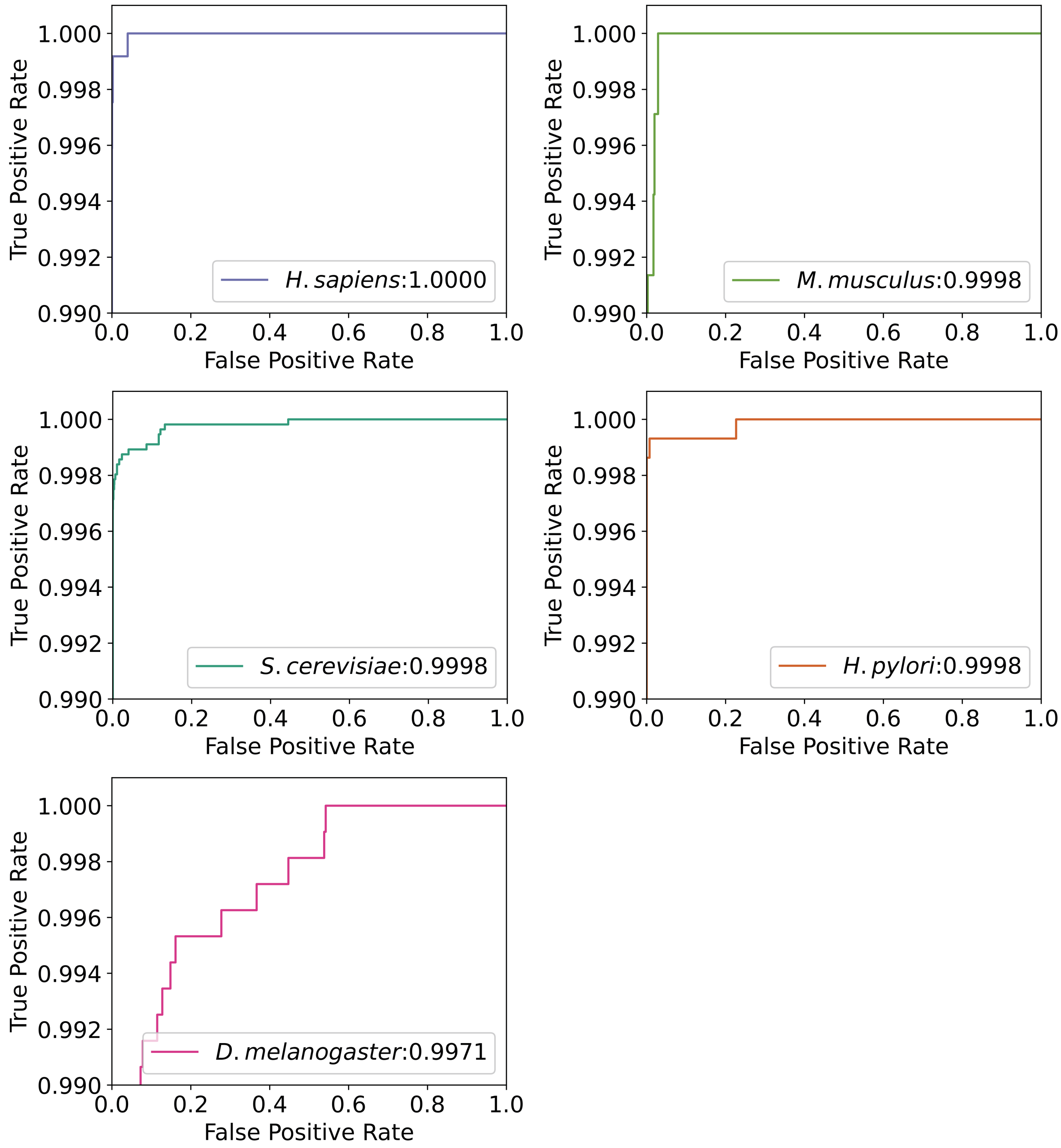

Supplementary Figure 1: The ROC-AUC curve of SVS models for protein-protein interaction identification. Note that all y-axes start from 0.990.

Supplementary Note 3 Model Optimization Parameters

Model	Parameters	choices	Remark
GBDT	n_estimators	10000	
	learning_rate	0.01	
	max_feature	'sqrt'	
	max_depth	i=7, j=8, k=9	i if train size < 1000
	min_samples_split	i=3, j=4, k=7	j if 1000≤train size<5000
	sbusample	i=0.7, j=0.5, k=0.3	k if train size ≥5000
ANN	N_layer	1,2,3,4,5,6	
	N_neuron	16, 32, 64, 128, 512, 1024	
	batch_size	32, 64, 128, 256	8, 16, 32 for small size datasets
	max_iter	20, 40, 80, 160, 320	
	alpha	0.01, 0.001, 0.0001, 0.00001	L2 regularization term
	learning_rate	0.01, 0.001, 0.0001	

Supplementary Table 2: GBDT and ANN parameters used in our study. All parameters not covered in the table use default values. We used Bayesian optimization to search the best combination of parameters for ANN models.

Supplementary Note 4 Datasets

In this section, we display overview of basic information and benchmark procedure for datasets used in our study in Table Supplementary Table 3. Then, we present our self-constructed datasets used for protein-protein and protein-nucleic acid binding affinity prediction. Finally, we show our selections of representations of PPI family for iPPI dataset.

S/N	name	source	train size	test size	Repetition
1	PL	PDBbind2016 [21]	3772	285	10
2	PP	PDBbind2020 [21]	1695	10-fold cross-validation	20
3	PN	PDBbind2020 [21]	186	10-fold cross-validation	20
4	iPPI	Rodrigues et al. [22]	1694	10-fold cross-validation	50
5	*S. cerevisiae*	Zhao et al. [2]	11188	5-fold cross-validation	1
6	*D. melanogaster*	Zhao et al. [2]	2140	5-fold cross-validation	1
7	*H. pylori*	Zhao et al. [2]	2916	5-fold cross-validation	1
8	*H. sapiens*	Zhao et al. [2]	2434	5-fold cross-validation	1
9	*M. musculus*	Zhao et al. [2]	694	5-fold cross-validation	1

Supplementary Table 3: Overview of basic information of all datasets used in this study. The number of repetitions follows the benchmark procedure of previous methods (if present).

Furthermore, we present the PP (protein-protein interaction) and PN (protein-nucleic acid interaction) datasets that we constructed in the task of binding affinity predictions. The original PDBbind2020 database provides the labels in term of dissociation constant (K_d), inhibitor constant (K_i), and half maximal inhibitory concentration (IC_{50}), we transform them into Gibbs free energy with the following equations:

$$\Delta G = -RTpK, \qquad \text{(Supplement Equation 8)}$$

where pK is $log_{10}K$ where K represents K_d or K_i. And IC_{50} can be approximately converted to K_i by the equation $K_i = IC_{50}/2$. R and T are the gas constant and temperature, respectively. At the room temperature, this formula becomes $\Delta G = -1.3633pK$.

PDB entry	label	ΔG	PDB entry	label	ΔG
1HVO	Kd=11uM	-6.76	1HVN	Kd=5uM	-7.227
1WET	Kd=1.5uM	-7.94	1BDH	Kd=900nM	-8.242
2PUF	Kd=2.0uM	-7.769	1QP4	Kd=10nM	-10.906
1QPZ	Kd=2.6nM	-11.704	1QQB	Kd=2000nM	-7.769
1QP0	Kd=35nM	-10.165	1QQA	Kd=400nM	-8.722
1QP7	Kd=780nM	-8.327	1DH3	Kd=1.4nM	-12.07
1FJX	Kd=1.4nM	-12.07	1JJ4	Kd=1.6nM	-11.991
1J75	Kd=35nM	-10.165	1FYM	Kd=40nM	-10.086
1JFS	Kd=2.2nM	-11.803	1JH9	Kd=2nM	-11.859
1J5K	Kd=3uM	-7.529	1JT0	Kd=49nM	-9.965
1PO6	Kd=157nM	-9.276	1P78	Kd=3.5nM	-11.528
1P51	Kd=3.5nM	-11.528	1P71	Kd=3.5nM	-11.528
1QZG	Kd=480nM	-8.614	1OMH	Kd=70nM	-9.754
1OSB	Kd=70nM	-9.754	1S40	Kd=0.3nM	-12.983
1TW8	Kd=1.14nM	-12.192	1U1L	Kd=107nM	-9.503
1U1P	Kd=209nM	-9.107	1U1R	Kd=229nM	-9.053
1U1M	Kd=296nM	-8.901	1U1N	Kd=316nM	-8.862
1U1K	Kd=319nM	-8.856	1U1O	Kd=59nM	-9.855
1U1Q	Kd=76nM	-9.706	2BJC	Kd=0.01nM	-14.996
2AOR	Kd=0.9uM	-8.242	1ZZI	Kd=2.2uM	-7.713
2AOQ	Kd=3.5uM	-7.438	2B0D	Kd=900nM	-8.242
2I9K	Kd=0.3nM	-12.983	2GII	Kd=0.42nM	-12.783
2GIJ	Kd=0.42nM	-12.783	2GIE	Kd=0.58nM	-12.592
2GIG	Kd=1.03nM	-12.252	2GIH	Kd=1.03nM	-12.252
2CCZ	Kd=100nM	-9.543	2ADY	Kd=14nM	-10.707
2AHI	Kd=20nM	-10.496	2AC0	Kd=25nM	-10.364
2ERG	Kd=25nM	-10.364	2AYB	Kd=2nM	-11.859
2AYG	Kd=2nM	-11.859	2ERE	Kd=30nM	-10.256
2ES2	Kd=326nM	-8.843	2GE5	Kd=381nM	-8.751
2ATA	Kd=84nM	-9.646	2O6M	Kd=13nM	-10.751
2NP2	Kd=55nM	-9.897	3D6Z	Kd=0.96uM	-8.204
3D6Y	Kd=10.3uM	-6.799	2VYE	Kd=46.9nM	-9.991
2VY1	Kd=95nM	-9.573	3IGM	Kd=0.5uM	-8.59
3HXO	Kd=0.6nM	-12.572	3HXQ	Kd=0.6nM	-12.572
3GIB	Kd=1.4nM	-12.07	3EQT	Kd=105nM	-9.514
3D2W	Kd=137.5nM	-9.355	3H15	Kd=3.4uM	-7.455
2KKF	Kd=44uM	-5.939	2KAE	Kd=8nM	-11.039
3M9E	Kd=0.13uM	-9.388	3MQ6	Kd=0.6nM	-12.572
3JSO	Kd=0.80nM	-12.402	3JSP	Kd=1.59nM	-11.995
3K3R	Kd=1.67nM	-11.966	3AAF	Kd=253nM	-8.994
3N1I	Kd=3.35nM	-11.554	3N1L	Kd=4.29nM	-11.407
3N1J	Kd=4.45nM	-11.386	3N1K	Kd=5.73nM	-11.236
3KJP	Kd=6.3nM	-11.18	3R8F	Kd=0.11uM	-9.487
3Q0B	Kd=1.08uM	-8.134	2RRA	Kd=1.5uM	-7.94
3PIH	Kd=1.9nM	-11.89	3QMI	Kd=12uM	-6.709
3QMH	Kd=17uM	-6.502	2KXN	Kd=2.25uM	-7.7

PDB entry	label	ΔG	PDB entry	label	ΔG
3QMB	Kd=3.5uM	-7.438	3ON0	Kd=4.8nM	-11.341
3U7F	Kd=600nM	-8.482	4HQU	Kd=0.02nM	-14.586
3RN2	Kd=0.112uM	-9.476	4HQX	Kd=1.2nM	-12.162
4ATK	Kd=1.7nM	-11.956	4A75	Kd=169nM	-9.232
2LTT	Kd=16nM	-10.628	3QSU	Kd=19.5nM	-10.511
4HIO	Kd=21nM	-10.467	4HJ8	Kd=22nM	-10.44
4HIK	Kd=24nM	-10.388	4HIM	Kd=37nM	-10.132
4A76	Kd=59nM	-9.855	4HJ5	Kd=63nM	-9.817
4HJ7	Kd=6nM	-11.209	4HP1	Kd=7uM	-7.028
4HID	Kd=855nM	-8.273	4J1J	Kd=0.505uM	-8.584
4LJ0	Kd=0.5uM	-8.59	4NM6	Kd=1.07uM	-8.14
4HT8	Kd=113nM	-9.471	4GCL	Kd=156.5nM	-9.278
4GCK	Kd=162.4nM	-9.256	3ZPL	Kd=2.4nM	-11.751
4F2J	Kd=25nM	-10.364	3ZH2	Kd=42nM	-10.057
4GCT	Kd=53.4nM	-9.915	4HT4	Kd=8nM	-11.039
4NI7	Kd=0.2nM	-13.223	4LNQ	Kd=1.33uM	-8.011
4CH1	Kd=249nM	-9.003	4LJR	Kd=30.6nM	-10.244
4QJU	Kd=370nM	-8.768	4R56	Kd=0.134uM	-9.37
4ZBN	Kd=0.21nM	-13.194	4TMU	Kd=1.5nM	-12.03
4R55	Kd=1.67uM	-7.876	4R22	Kd=11.6nM	-10.819
4S0N	Kd=13nM	-10.751	4Z3C	Kd=2.4uM	-7.661
4ZSF	Kd=2.9nM	-11.639	3WPC	Kd=20nM	-10.496
4RKG	Kd=25.7uM	-6.258	3WPD	Kd=3nM	-11.619
5A72	Kd=53.3nM	-9.916	2N8A	Kd=95nM	-9.573
5DWA	Kd=0.9nM	-12.332	5HRT	Kd=1.3nM	-12.114
5K83	Kd=1.57uM	-7.913	5T1J	Kd=19nM	-10.526
5ITH	Kd=0.03uM	-10.256	5VC9	Kd=0.16uM	-9.265
5W9S	Kd=0.1uM	-9.543	5HLG	Kd=0.31uM	-8.873
5YI3	Kd=0.55uM	-8.534	5W9Q	Kd=0.6uM	-8.482
5XFP	Kd=1.2uM	-8.072	6ASB	Kd=1.5uM	-7.94
5MEY	Kd=160nM	-9.265	6ASD	Kd=2.4uM	-7.661
5MEZ	Kd=250nM	-9.001	5K07	Kd=32.3uM	-6.122
5W2M	Kd=6.36uM	-7.084	5YI2	Kd=72nM	-9.738
5K17	Kd=8.82uM	-6.891	6MG3	Kd=0.004uM	-11.449
6MG1	Kd=0.029uM	-10.276	6FWR	Kd=0.31uM	-8.873
5VMV	Kd=0.43nM	-12.769	6CNP	Kd=0.7uM	-8.391
6CNQ	Kd=0.9uM	-8.242	5ZD4	Kd=12nM	-10.798
6FQP	Kd=169.9nM	-9.229	5ZMO	Kd=190nM	-9.163
5WWF	Kd=238.1nM	-9.029	6BWY	Kd=26uM	-6.251
6FQQ	Kd=388.6nM	-8.739	6CRM	Kd=4.7uM	-7.264
5ZVB	Kd=40.38uM	-5.99	6CC8	Kd=5.4uM	-7.181
5ZVA	Kd=54.86uM	-5.809	6BUX	Kd=55uM	-5.807
5MPF	Kd=90nM	-9.605	6A2I	Kd=0.415uM	-8.701
6G1L	Kd=0.9nM	-12.332	5ZKI	Kd=1.32uM	-8.015
6IIQ	Kd=1.3uM	-8.024	6KBS	Kd=2.2uM	-7.713
6IIR	Kd=2.8uM	-7.57	5ZKL	Kd=20.6nM	-10.479

PDB entry	label	ΔG	PDB entry	label	ΔG
5ZMD	Kd=3.69uM	-7.407	6ON0	Kd=90nM	-9.605

Supplementary Table 4: The PDB entries, labels, and the corresponce Gabbs free enegy(ΔG) of the PN (protein-nucleic acid interaction) dataset used in our study. This dataset was constructed based on protein-nucleic acid complexes of PDBbind2020.

PDB entry	label	ΔG	PDB entry	label	ΔG
2NYZ	Kd=0.9nM	-12.332	3OAK	Kd=169nM	-9.232
2ZXX	Kd=4.4nM	-11.392	4U2X	Kd=1nM	-12.27
3BRV	IC50=47nM	-9.99	2FDB	Kd=155nM	-9.284
4Z80	Kd=12nM	-10.798	3M63	Kd=175nM	-9.212
4A49	Kd=42uM	-5.967	3ETB	Kd=29pM	-14.366
2Q5D	Kd=15.6nM	-10.643	1E96	Kd=2.7uM	-7.592
5B75	Kd=10uM	-6.816	5YIP	Kd=3.7nM	-11.495
1BUH	Kd=77nM	-9.698	3C4M	Kd=0.98uM	-8.192
5KOV	Kd=1.87nM	-11.899	5Z7L	Kd=1.2uM	-8.072
5EG3	Kd=0.135uM	-9.365	6H16	Kd=31.8nM	-10.221
6FV0	Kd=1.72uM	-7.859	4YIZ	Kd=8nM	-11.039
5DCQ	Kd=0.18uM	-9.195	2WH6	Kd=18nM	-10.558
4UEM	Kd=6.4nM	-11.171	5KY5	Kd=0.79uM	-8.319
2ZVO	Kd=1.6uM	-7.902	4U32	Ki=379nM	-8.754
5HVF	Kd=0.03nM	-14.346	1ACB	Kd=0.2nM	-13.223
5AYS	Kd=0.508nM	-12.671	1GCQ	Kd=16.8uM	-6.509
3C9A	Kd=7.7nM	-11.061	5HLZ	Kd=5nM	-11.317
5V52	Kd=3.7uM	-7.405	2M86	Kd=2.6nM	-11.704
1MAH	Kd=25pM	-14.454	4ZI2	Kd=0.42uM	-8.693
5IIA	Kd=1.81nM	-11.918	2IYB	Kd=3.6uM	-7.421
4XKL	Kd=39.1uM	-6.009	6IDX	Kd=3.36uM	-7.462
2Y5B	Kd=4.2uM	-7.33	4Z9K	Kd=2.2nM	-11.803
3ONW	Kd=2.3nM	-11.777	4LXR	Kd=5.9nM	-11.219
4WEM	Kd=6.8uM	-7.045	2OT8	Kd=20nM	-10.496
5ML9	Kd=217nM	-9.084	5MTJ	Kd=338nM	-8.822
6JHW	Kd=18nM	-10.558	1PVH	Kd=80nM	-9.675
5IMT	Kd=367nM	-8.773	2Z34	Kd=0.89uM	-8.249
6FUD	Kd=73nM	-9.729	4GLA	Kd=35.3uM	-6.07
1F5R	Ki=65uM	-5.708	5W3X	Kd=3.37uM	-7.46
3D5O	Kd=1uM	-8.18	3QML	Kd=13nM	-10.751
6OV2	Kd=3.58nM	-11.515	4C99	Kd=114nM	-9.466
1FLT	Kd=1.356nM	-12.089	5GWP	Kd=2.1nM	-11.83
2JJS	Kd=1.2uM	-8.072	6FQ0	Kd=160nM	-9.265
6IUA	Kd=2.0nM	-11.859	3ZS9	Kd=150nM	-9.303
3F5C	Kd=0.9uM	-8.242	2YQ7	Ki=35nM	-10.165
6GUM	Kd=1.2uM	-8.072	5NVK	Kd=0.4nM	-12.812
4U30	Ki=138nM	-9.352	4NU1	Ki=60uM	-5.756
3TAC	Kd=0.55uM	-8.534	6MGP	Kd=680nM	-8.408
2C5D	Kd=6nM	-11.209	4M38	Kd=12uM	-6.709
6GBG	Kd=417nM	-8.698	5KY0	Kd=6.5uM	-7.072
5YI8	Kd=1.8uM	-7.832	2BYK	Kd=2.3uM	-7.687
3C4O	Kd=4.6nM	-11.366	5LRW	Kd=9.3uM	-6.859
4G59	Kd=0.42uM	-8.693	1SMF	Ki=0.12uM	-9.435
4B1Y	Kd=0.27uM	-8.955	2F9Z	Kd=0.9uM	-8.242
4X33	Kd=0.25uM	-9.001	5TZN	Kd=5.8uM	-7.139
3SJD	Kd=31nM	-10.237	2ONL	Kd=6nM	-11.209

PDB entry	label	ΔG	PDB entry	label	ΔG
5M2J	Kd=0.13nM	-13.478	5OYL	Kd=7.5uM	-6.987
2IWG	Kd=37nM	-10.132	2WX0	Kd=8uM	-6.949
6I2M	Kd=5.31nM	-11.281	6FU9	Kd=4.7nM	-11.353
4RS1	Kd=23.9nM	-10.391	1XQS	Kd=6.5uM	-7.072
2LKM	Kd=12nM	-10.798	6F0F	Kd=0.18uM	-9.195
3KJ1	Kd=2nM	-11.859	3DXE	Kd=0.33uM	-8.836
1VRC	Kd=28.4uM	-6.198	2KGX	Kd=9uM	-6.879
5OTX	IC50=187nM	-9.172	3IDC	Kd=0.6nM	-12.572
1KXP	Kd=1nM	-12.27	4RJF	Kd=250nM	-9.001
1FLE	Ki=6nM	-11.209	6DWF	Ki=1.3nM	-12.114
5CTR	Kd=0.88uM	-8.255	6C83	Kd=1.0uM	-8.18
3OLM	Kd=90.6uM	-5.512	3BIW	Kd=97nM	-9.561
2QJB	Kd=4.9nM	-11.329	3OUX	Kd=35nM	-10.165
2K8F	Kd=2.7uM	-7.592	3REB	Kd=96nM	-9.567
2PMS	Kd=10.3nM	-10.889	4EUK	Kd=4.1uM	-7.344
4C2B	Kd=9nM	-10.969	4QT8	Kd=0.28uM	-8.933
3DI3	Kd=21nM	-10.467	3V4Y	Kd=17.8nM	-10.565
5EOF	Kd=3.8uM	-7.389	4PBZ	Kd=0.05uM	-9.953
4JMF	Kd=0.298uM	-8.897	1VEU	Kd=12.8nM	-10.76
2BTF	Kd=2.3uM	-7.687	5G1X	Kd=12.1uM	-6.704
4EQA	Kd=2.42nM	-11.746	1ZC3	Kd=42nM	-10.057
5O90	Kd=1.1uM	-8.123	5EP6	Kd=0.72uM	-8.374
2BO9	Ki=3nM	-11.619	3UJG	IC50=1.7uM	-7.866
1NYS	Kd=10nM	-10.906	3ZWZ	Kd=20.3nM	-10.487
4J32	Kd=1.28nM	-12.124	5U52	Kd=27nM	-10.318
1F3V	Kd=7.8uM	-6.964	1TM7	Ki=0.0033nM	-15.653
1YC0	IC50=4nM	-11.449	5MFC	Kd=18nM	-10.558
5NT1	Kd=16.1uM	-6.535	3ZS8	Kd=51nM	-9.942
3SYN	Kd=0.6uM	-8.482	1SYQ	Kd=14.7nM	-10.678
3QT2	Kd=1.9nM	-11.89	2LFW	Kd=5.6nM	-11.25
3AON	Kd=3.2nM	-11.581	4LYL	Kd=31nM	-10.237
4PGJ	Kd=26nM	-10.341	4NG2	Kd=3.2uM	-7.491
2O8V	Ki=1.1uM	-8.123	4EXP	Kd=500nM	-8.59
4V0O	Kd=348nM	-8.805	4ZW2	Kd=4.9nM	-11.329
4PZ6	Kd=0.21uM	-9.104	1DZB	Kd=520nM	-8.567
4U5W	Kd=2.63uM	-7.607	2JU0	Kd=100nM	-9.543
3HTU	Kd=1.8uM	-7.832	2QIY	Kd=119nM	-9.44
1T63	Kd=15uM	-6.576	2C7M	Kd=6.4uM	-7.081
1RY7	Kd=0.23uM	-9.05	2J59	Kd=55nM	-9.897
4JE4	Kd=14nM	-10.707	4XL1	Kd=304nM	-8.885
6CXL	Kd=2.4nM	-11.751	6H9N	Kd=0.8uM	-8.312
2KDE	Kd=8.9uM	-6.885	2Z3Q	Kd=38pM	-14.206
6SAK	Kd=0.031uM	-10.237	3C4P	Kd=4.4nM	-11.392
4LLO	Kd=13.2uM	-6.652	5KXH	Kd=0.49uM	-8.602
5D1L	Kd=141nM	-9.34	1M5N	Kd=2.1nM	-11.83
4M0W	Kd=6.7uM	-7.054	4KSD	IC50=520nM	-8.567

PDB entry	label	ΔG	PDB entry	label	ΔG
5NUS	Kd=11nM	-10.85	5XBF	Kd=1.7uM	-7.866
5Y4R	Kd=28.6nM	-10.284	3UYO	Kd=6.8uM	-7.045
2KWV	Kd=90uM	-5.516	3P9W	Kd=16nM	-10.628
6IRD	Kd=0.21uM	-9.104	5NQF	Kd=169nM	-9.232
5HU3	Kd=1uM	-8.18	2Z7F	IC50=13nM	-10.751
2ZVK	Kd=0.4uM	-8.722	6A6X	Kd=25.8nM	-10.345
2X89	Kd=44nM	-10.029	5W89	IC50=25nM	-10.364
1GXD	Kd=5.2nM	-11.294	5MAK	Kd=29pM	-14.366
1YX6	Kd=73uM	-5.64	3GJ7	Kd=4uM	-7.359
4LRX	Kd=15uM	-6.576	1J7V	Kd=35pM	-14.255
2EFH	Kd=36nM	-10.148	3ZUV	Kd=117nM	-9.45
3DOE	Kd=20nM	-10.496	4C2A	Kd=9.7nM	-10.924
3QBR	IC50=68nM	-9.771	4C4K	Kd=2.7uM	-7.592
5O2T	Kd=167nM	-9.239	3FIE	Ki=1nM	-12.27
3D5S	Kd=26nM	-10.341	5FVK	Kd=1.8uM	-7.832
6BXC	Kd=13nM	-10.751	3HCU	Kd=1.48uM	-7.948
3ALZ	Kd=0.52uM	-8.567	5VX0	Kd=21uM	-6.377
4BPK	IC50=25nM	-10.364	3W8H	Kd=0.164uM	-9.25
1MQ8	Kd=3uM	-7.529	1XR0	Kd=10uM	-6.816
6BMT	Kd=310nM	-8.873	1B6C	Kd=0.28uM	-8.933
5UZU	Kd=10nM	-10.906	6JB8	Kd=0.129nM	-13.482
2SNI	Kd=2pM	-15.949	1SBB	Kd=140uM	-5.254
4AYD	Kd=820nM	-8.297	4ZKC	Kd=0.22nM	-13.166
5LP5	Kd=0.4uM	-8.722	5JKE	Kd=91nM	-9.599
6FC2	Kd=3400nM	-7.455	2RF9	Kd=13uM	-6.661
4XHU	Kd=26nM	-10.341	1R0R	Kd=29.4pM	-14.358
5CXB	Kd=9.19nM	-10.956	4FT4	Kd=0.5uM	-8.59
6G04	Kd=0.7uM	-8.391	2VLN	Kd=1.68pM	-16.052
3WN7	Kd=7.31nM	-11.092	1JGN	Kd=0.35uM	-8.801
5E95	Kd=15.7nM	-10.639	3UIR	Ki=0.436nM	-12.761
4L0P	Kd=9nM	-10.969	1A2K	Kd=150nM	-9.303
3CQC	Kd=4nM	-11.449	5DOB	Kd=1uM	-8.18
4W6W	Kd=3.57nM	-11.516	5TAR	Kd=2.3uM	-7.687
1AXI	Kd=14nM	-10.707	4HDO	Kd=0.36uM	-8.785
6D4P	IC50=65nM	-9.798	3K8P	Kd=10nM	-10.906
5BN5	Kd=11uM	-6.76	4APF	Kd=0.65uM	-8.435
3QSK	Kd=91nM	-9.599	3RDZ	Kd=2.69nM	-11.684
5CMN	Kd=220nM	-9.076	1DFJ	Ki=59fM	-18.035
3WRY	Kd=0.31uM	-8.873	1T01	Kd=39nM	-10.101
2NBV	Kd=9.3uM	-6.859	3ZET	Kd=0.29uM	-8.913
1KGY	Kd=25nM	-10.364	1XXF	IC50=200nM	-9.133
1S4Y	Kd=6.87nM	-11.129	4WWI	Kd=15.2nM	-10.658
1EWY	Kd=3.57uM	-7.426	2HQS	Kd=27nM	-10.318
4DI3	Kd=0.06uM	-9.846	1P69	Kd=76nM	-9.706
1GO4	Kd=1.04uM	-8.157	5Z2W	Kd=0.67uM	-8.417
3DVU	Kd=0.2uM	-9.133	6EJG	Kd=0.81nM	-12.394

PDB entry	label	ΔG	PDB entry	label	ΔG
3QTL	Ki=1.43nM	-12.058	1VET	Kd=12.8nM	-10.76
5IMK	Kd=850nM	-8.276	5KWY	Kd=429nM	-8.681
5AOQ	Kd=4.1nM	-11.434	3OUW	Kd=23nM	-10.413
2B4J	Kd=10.9nM	-10.855	2K42	Kd=35nM	-10.165
1G6V	Kd=72nM	-9.738	2JT4	Kd=40uM	-5.996
5AAM	Kd=3.8nM	-11.479	6JWJ	Kd=85.7nM	-9.634
2LZ6	Kd=12uM	-6.709	4F38	Kd=2.9nM	-11.639
4H6J	Kd=190nM	-9.163	4APX	Kd=2.9uM	-7.549
3FII	Ki=1.9nM	-11.89	4PP8	Kd=486nM	-8.607
5AAW	Kd=4.32nM	-11.403	4LN0	Kd=6.8nM	-11.135
6DWH	Ki=0.2nM	-13.223	2MEJ	Kd=17uM	-6.502
6J4S	Kd=0.16uM	-9.265	1J2J	Kd=1.4uM	-7.981
4HFF	Kd=0.269nM	-13.047	2C0L	Kd=109nM	-9.492
2LD7	Kd=9.2nM	-10.956	2VLO	Kd=0.892pM	-16.427
4LZX	Kd=5.07nM	-11.309	5VZM	Kd=48.8uM	-5.878
3IXS	Kd=90nM	-9.605	4H5S	Kd=12.5uM	-6.684
5WB7	Kd=0.76uM	-8.342	1BVN	Ki=9pM	-15.059
4C9B	Kd=28.4nM	-10.288	3T1Q	Kd=5uM	-7.227
2I32	Kd=1.3uM	-8.024	3SJA	Kd=17nM	-10.592
1TDQ	Kd=12nM	-10.798	2IOU	Kd=3.47uM	-7.443
4HSA	Kd=1.9nM	-11.89	1Y4D	Ki=1.2nM	-12.162
5BNQ	Kd=0.156nM	-13.37	4ZGQ	Kd=2.5uM	-7.637
2OMZ	Kd=400nM	-8.722	1AVA	Ki=0.22nM	-13.166
2LP0	Kd=22.2uM	-6.344	3BLH	Kd=300nM	-8.893
3C59	IC50=5nM	-11.317	1X1X	Kd=53nM	-9.919
6GHO	Kd=0.8uM	-8.312	4FZA	Kd=99.1nM	-9.548
5GY2	IC50=204pM	-13.211	5NT7	Kd=10.6uM	-6.782
3PNR	Ki=11.9nM	-10.803	3OXU	Kd=34uM	-6.092
4G35	Ki=54nM	-9.908	5KOF	Kd=37.6uM	-6.032
6HT9	Ki=0.1nM	-13.633	4M6B	Kd=7.1nM	-11.109
1RV6	IC50=275nM	-8.944	2C1T	Kd=2.0nM	-11.859
1ZGU	Kd=98uM	-5.465	2KWI	Kd=184nM	-9.182
4NM3	Ki=60uM	-5.756	3VUX	Kd=9uM	-6.879
2IXQ	Kd=0.7uM	-8.391	3N00	IC50=0.22uM	-9.076
6IHB	Kd=57nM	-9.876	3WDG	Kd=1.198nM	-12.163
5VKO	Kd=4nM	-11.449	1E6E	Kd=0.86uM	-8.269
2RMS	Kd=134nM	-9.37	3RGF	Kd=7.05nM	-11.113
2FTM	Kd=16pM	-14.718	3DXC	Kd=0.22uM	-9.076
3UL4	Kd=11.4nM	-10.829	2IJ0	Kd=180pM	-13.285
3KBT	Kd=9.29nM	-10.95	4AOR	Kd=60.9nM	-9.837
2VDB	Kd=0.15nM	-13.393	4ZRJ	Kd=2.9uM	-7.549
2KYG	Kd=67nM	-9.78	2JOD	Ki=350nM	-8.801
2JJT	Kd=1.2uM	-8.072	4UWX	Kd=6.9uM	-7.036
5LGK	Kd=1.2uM	-8.072	2V3B	Kd=5uM	-7.227
4J2L	Kd=88.5nM	-9.615	2IBF	Kd=7.8nM	-11.054
4DGE	Kd=38.5uM	-6.018	1HE8	Kd=2.5uM	-7.637

PDB entry	label	ΔG	PDB entry	label	ΔG
4YPI	Kd=19.4nM	-10.514	4W4O	Kd=4.2nM	-11.42
3SF4	Kd=2.4nM	-11.751	5MV9	Kd=1uM	-8.18
3ZO0	Kd=437nM	-8.67	5EE5	Kd=26uM	-6.251
3EJJ	Kd=455nM	-8.646	6EG0	Kd=77.7uM	-5.603
3INB	Kd=0.2uM	-9.133	5O05	Kd=0.81nM	-12.394
6IRE	Kd=10.6nM	-10.872	4F9P	Kd=15nM	-10.666
1D6R	Kd=0.13nM	-13.478	6PLM	Kd=89.6nM	-9.608
3MJ7	Kd=5uM	-7.227	1DP5	Ki=0.9nM	-12.332
5AJJ	Kd=3220nM	-7.487	1SB0	Kd=15uM	-6.576
2VAY	Kd=7.9nM	-11.046	4HH3	Kd=1.3uM	-8.024
4BKX	Kd=5uM	-7.227	2JBY	IC50=50nM	-9.953
4POU	Kd=157nM	-9.276	2P48	Kd=116nM	-9.455
2Y8S	Kd=0.989nM	-12.276	2QNA	Kd=83nM	-9.653
3C66	Kd=3uM	-7.529	1USV	Kd=3.3uM	-7.473
2OTK	Kd=17nM	-10.592	3QWQ	Kd=2nM	-11.859
4X7S	Kd=10nM	-10.906	6H46	Kd=30nM	-10.256
1AN1	Ki=0.9nM	-12.332	2GWW	Kd=0.11nM	-13.577
6FF3	Kd=13.6nM	-10.724	2N73	Kd=320nM	-8.854
1Y6M	Kd=173nM	-9.219	4YOC	Kd=0.6uM	-8.482
6A3V	Kd=85.6nM	-9.635	5INB	Kd=123nM	-9.421
5YIR	Kd=0.27nM	-13.045	5B77	Kd=10.8uM	-6.771
4EKC	Kd=22nM	-10.44	3OL2	Kd=5.5uM	-7.17
1Y6N	Kd=27nM	-10.318	4WEN	Kd=0.1uM	-9.543
1DHK	Ki=35pM	-14.255	2B12	Kd=20uM	-6.406
5ODW	Kd=240pM	-13.115	4X7D	Kd=3.5nM	-11.528
6NE2	Kd=1.3nM	-12.114	6K06	Kd=227nM	-9.058
3FJU	Ki=1.6nM	-11.991	2RMK	Kd=170nM	-9.229
6BXA	Kd=40nM	-10.086	6F0H	Kd=3nM	-11.619
3SGK	Kd=2.9nM	-11.639	4IC7	Kd=0.43uM	-8.679
4DKD	Kd=94nM	-9.58	1IBR	Kd=0.3nM	-12.983
1VSQ	Kd=0.5mM	-4.5	5CHV	Kd=1.49uM	-7.944
5YIS	Kd=4.2nM	-11.42	2MCN	Kd=1.9uM	-7.8
2J8X	Ki=8nM	-11.039	3K9M	Ki=2nM	-11.859
4JZW	Kd=15.4pM	-14.741	2WY3	Kd=66nM	-9.789
6OQJ	Kd=9nM	-10.969	1I4E	Kd=0.12uM	-9.435
1RI8	Kd=2.9nM	-11.639	2F4M	Kd=65nM	-9.798
1ZHI	Kd=0.2uM	-9.133	3OWT	Kd=2.49uM	-7.64
3N0P	Kd=5.1mM	-3.125	3NPZ	Kd=3.15uM	-7.5
2MJW	Kd=6.2uM	-7.1	5E7F	Kd=9.1nM	-10.962
1TM3	Ki=0.019nM	-14.616	4ML7	Kd=0.27nM	-13.045
5LZ6	Kd=8uM	-6.949	3TGK	Ki=12uM	-6.709
2AQ3	Kd=7.6uM	-6.979	1ZM4	Kd=0.98uM	-8.192
4PBV	Kd=2.4uM	-7.661	5XEQ	Kd=0.4nM	-12.812
6BX8	Ki=0.2uM	-9.133	5M2M	Kd=1.5nM	-12.03
1OQE	Kd=109nM	-9.492	2W19	Kd=140nM	-9.344
2XDF	Kd=21uM	-6.377	5XJG	Kd=4.52uM	-7.287

PDB entry	label	ΔG	PDB entry	label	ΔG
6GFI	Ki=494pM	-12.687	6ISC	Kd=2.4uM	-7.661
2ZVN	Kd=1.6uM	-7.902	2WY8	Kd=360nM	-8.785
2UYZ	Kd=82nM	-9.661	6EG1	Kd=76.6uM	-5.611
2K3U	Kd=8.4nM	-11.01	4JEG	Kd=12nM	-10.798
3R4D	Kd=21.4nM	-10.456	6FPG	Kd=101.9nM	-9.532
3TKL	Kd=0.43uM	-8.679	3H3G	Kd=0.99uM	-8.186
5OMM	Kd=10.4nM	-10.883	5ZO2	Kd=1.1uM	-8.123
6OSW	Kd=2uM	-7.769	5TP6	Kd=1.9nM	-11.89
4UI1	Kd=124nM	-9.416	4HRL	Kd=1nM	-12.27
6CZO	Kd=89nM	-9.612	5IXD	Kd=70.5nM	-9.75
4MYW	Kd=19.1nM	-10.523	6OBN	Kd=30.4nM	-10.248
2KWJ	Kd=0.5uM	-8.59	2OOR	Kd=15.5nM	-10.647
4DBG	Kd=0.52uM	-8.567	5V5G	Kd=9nM	-10.969
1L4D	Kd=196.6nM	-9.143	1WQ1	Kd=17uM	-6.502
4G8A	Kd=186pM	-13.266	6FHP	Kd=48nM	-9.978
3CQX	Kd=4.5uM	-7.289	2WO2	Kd=10.8uM	-6.771
2OZA	Kd=2.5nM	-11.727	1JTG	Ki=0.11nM	-13.577
6O3O	Kd=1.8uM	-7.832	1Y76	Kd=5uM	-7.227
4NL9	Kd=249nM	-9.003	2FU5	Kd=0.7nM	-12.481
2QUR	IC50=500nM	-8.59	2WO3	Kd=2.3uM	-7.687
2J8S	Kd=28nM	-10.297	4F9L	Kd=66nM	-9.789
6IWA	Kd=126nM	-9.406	1Y48	Ki=1.7nM	-11.956
6H47	Kd=10nM	-10.906	1FG9	Kd=0.10nM	-13.633
2WQZ	Kd=132nM	-9.379	4A1U	Ki=0.54uM	-8.545
4BWQ	Kd=19.7uM	-6.415	2J1K	Kd=1.1nM	-12.213
4CJ0	Kd=98nM	-9.555	5V6A	Kd=53.2nM	-9.917
5MTM	Kd=7.0nM	-11.118	3KJ2	Kd=2nM	-11.859
5J28	Kd=193nM	-9.154	4B1V	Kd=0.32uM	-8.854
5JZE	Kd=7.48uM	-6.988	3QBT	Kd=0.9uM	-8.242
2LEH	Kd=3.3nM	-11.563	4H2S	Kd=21.5uM	-6.363
4IW4	Kd=610nM	-8.472	4NZL	Kd=25nM	-10.364
1EJA	Ki=1.0nM	-12.27	5KY4	Kd=10uM	-6.816
3UZV	Kd=0.43nM	-12.769	3Q4F	Kd=4.1uM	-7.344
5ABV	Kd=0.52nM	-12.657	5XOC	Kd=8uM	-6.949
2VOF	Kd=0.7nM	-12.481	4GU0	Kd=0.741uM	-8.357
6BW9	Kd=4.39nM	-11.394	5BW7	Kd=52.3nM	-9.927
2WP0	Kd=0.93uM	-8.223	2KWO	Kd=46.9uM	-5.901
1KTK	Kd=20uM	-6.406	3F50	Ki=9nM	-10.969
1LJ2	Kd=27uM	-6.228	4BD9	Ki=31nM	-10.237
3MCA	Kd=0.39uM	-8.737	4RWT	Kd=0.23uM	-9.05
1XU1	IC50=5.9nM	-11.219	6JCS	Kd=0.287uM	-8.919
1M10	Kd=5.8nM	-11.229	2MV7	Kd=1.56nM	-12.006
1GL1	Ki=0.13nM	-13.478	3V6B	Kd=0.28uM	-8.933
4BYF	Kd=0.9uM	-8.242	2N1D	Kd=60nM	-9.846
5C3F	IC50=1.6uM	-7.902	2I26	Kd=9.4nM	-10.943
2PCB	Kd=10uM	-6.816	3O40	Ki=1.4nM	-12.07

PDB entry	label	ΔG	PDB entry	label	ΔG
3G7A	Ki=9nM	-10.969	1Z92	Kd=10nM	-10.906
4LQW	Kd=94uM	-5.49	4PBW	Kd=551nM	-8.533
5BOZ	Kd=0.7nM	-12.481	6PNQ	Kd=49nM	-9.965
5VT9	Kd=10nM	-10.906	3ZWH	Kd=1540nM	-7.924
4DPG	Kd=90nM	-9.605	5J7C	Kd=0.209nM	-13.197
1T0P	Kd=25uM	-6.274	4IHH	Kd=59nM	-9.855
1WQJ	Kd=865nM	-8.266	4ZQU	Kd=16nM	-10.628
3R9A	Kd=3.5uM	-7.438	5YDR	Kd=26.4uM	-6.242
5UN7	Kd=120nM	-9.435	2M0G	Kd=84nM	-9.646
3RO2	Kd=0.046uM	-10.003	3EOY	Kd=2.8nM	-11.66
3EHU	IC50=6.6uM	-7.063	4XVP	Kd=4.2nM	-11.42
4QLB	Kd=1.6uM	-7.902	5VKL	Kd=113nM	-9.471
2LNK	Kd=4nM	-11.449	2PCC	Kd=1.6uM	-7.902
5VX2	Kd=2.5nM	-11.727	1MZW	Kd=1.97uM	-7.778
1SV0	Kd=11.1nM	-10.845	4KBB	Kd=0.18uM	-9.195
2I9B	Kd=0.96nM	-12.294	4V2C	Kd=18uM	-6.468
6H71	Kd=43.4nM	-10.037	3MJH	Kd=2.4uM	-7.661
2UUY	Ki=5.6nM	-11.25	4U0Q	Kd=1.1uM	-8.123
2IY1	Kd=787nM	-8.322	4A1S	Kd=5nM	-11.317
4IYP	Kd=0.3uM	-8.893	5UUL	Ki=4.8nM	-11.341
3SGB	Kd=17.9pM	-14.652	2K6D	Kd=171uM	-5.136
2HTH	Kd=105uM	-5.424	5MJY	Kd=11.3uM	-6.744
1H1V	Kd=23nM	-10.413	4AN7	Ki=3.2nM	-11.581
4NSO	Kd=0.98nM	-12.282	2X1X	Kd=16nM	-10.628
2FUH	Kd=300uM	-4.803	5TL7	Kd=1870uM	-3.719
2V4Z	Kd=1.25uM	-8.048	3NOG	Kd=22.4nM	-10.429
6OAN	IC50=4.88nM	-11.331	4ZRK	Kd=1uM	-8.18
3BN3	Kd=20uM	-6.406	4YWC	IC50=0.49uM	-8.602
4PJ2	Kd=47pM	-14.08	1OTR	Kd=155uM	-5.194
4DM8	Kd=1.5uM	-7.94	5MA3	Kd=38pM	-14.206
5D3F	Kd=8.05uM	-6.945	1DPJ	Ki=3nM	-11.619
4DG4	Ki=2.7uM	-7.592	2X9A	Kd=4.4uM	-7.303
4EKD	Kd=22nM	-10.44	5E3E	Kd=40.9nM	-10.072
4XOI	Kd=7.3uM	-7.003	3AU4	Kd=0.53uM	-8.556
3E2K	Ki=69pM	-13.853	1Y8N	Kd=1.17uM	-8.087
5XWT	Kd=14.4uM	-6.601	2KJ4	Kd=65nM	-9.798
5J4A	Kd=72nM	-9.738	4OJK	Kd=22uM	-6.35
1T44	Kd=1uM	-8.18	4WEU	Kd=5.7uM	-7.149
5HPK	Kd=9.7nM	-10.924	6E3I	Kd=3.21nM	-11.579
3L33	Ki=136nM	-9.361	4KGG	Kd=153nM	-9.291
4J6G	Kd=16.3nM	-10.617	3F1P	Kd=120uM	-5.345
1YK1	Kd=45.7nM	-10.007	3OED	Kd=0.5uM	-8.59
1L0X	Kd=6uM	-7.119	5SZI	Kd=253nM	-8.994
5UV8	Kd=140.6nM	-9.341	3FF8	Kd=130uM	-5.298
3TG1	Ki=1.01uM	-8.174	6JDJ	Kd=200nM	-9.133
3H8K	Kd=21nM	-10.467	2K05	Kd=0.2uM	-9.133

PDB entry	label	ΔG	PDB entry	label	ΔG
1KBH	Kd=34nM	-10.182	6AMB	Kd=17.8uM	-6.475
3VHX	Kd=15uM	-6.576	5GJK	Kd=0.12uM	-9.435
4EAH	IC50=1.7nM	-11.956	3CX6	Kd=4.8uM	-7.251
5NWM	Kd=0.04uM	-10.086	1YD8	Kd=181uM	-5.102
6FTO	Kd=2.6nM	-11.704	5TL6	Kd=57.6uM	-5.78
3HO5	Kd=220nM	-9.076	2K8C	Kd=1.8mM	-3.742
6MUN	Kd=1uM	-8.18	2A9K	Kd=60nM	-9.846
1JH4	Kd=1.5uM	-7.94	4KVG	Kd=0.7uM	-8.391
6D13	Kd=9.3nM	-10.949	4UDM	Kd=4nM	-11.449
3NVQ	Kd=287nM	-8.919	3DGC	Kd=1nM	-12.27
1PJN	Kd=22nM	-10.44	5UA4	Kd=26nM	-10.341
6FE4	Kd=9.6nM	-10.931	2GH0	Kd=90nM	-9.605
4TQ0	Kd=6.9uM	-7.036	3KLS	Kd=5.5nM	-11.26
4PW9	Kd=13.5uM	-6.639	6NRW	Kd=23uM	-6.323
4DXA	Kd=1.8uM	-7.832	2XNS	Kd=810nM	-8.305
2P44	Kd=20nM	-10.496	3BP8	Kd=4.14nM	-11.429
3NOC	Kd=1.5nM	-12.03	4PAS	Kd=126.6nM	-9.403
5VWX	Kd=14.9nM	-10.67	5GPG	IC50=2.61nM	-11.702
4ZV4	Kd=23nM	-10.413	5O03	Kd=60nM	-9.846
3EG5	Kd=1253nM	-8.046	5V5H	Kd=158nM	-9.272
2HEV	IC50=62nM	-9.826	1F7Z	Ki=12.4uM	-6.689
4XLW	Kd=262nM	-8.973	4ZLT	Kd=1.4nM	-12.07
4EIG	Kd=1nM	-12.27	5T0F	IC50=58nM	-9.866
6IMF	Kd=24nM	-10.388	4AYE	Kd=3330nM	-7.468
5FR2	Kd=141nM	-9.34	1TE1	Ki=3.4nM	-11.545
4RT6	Kd=13.2nM	-10.742	1SQ0	Kd=30nM	-10.256
2QHO	Kd=60uM	-5.756	2XQR	Kd=31nM	-10.237
2KNB	Kd=11uM	-6.76	4EHQ	Kd=1.3uM	-8.024
5JDS	Kd=3nM	-11.619	4JW3	Kd=1450nM	-7.96
6OG4	Kd=1.29nM	-12.119	6UYS	Kd=1.6uM	-7.902
3H2U	Kd=12.6uM	-6.68	4OFY	Kd=0.602uM	-8.48
5IBW	Kd=0.25nM	-13.09	4YIQ	Kd=2uM	-7.769
1B2U	Kd=100nM	-9.543	1KKL	Kd=45nM	-10.016
1EZU	Ki=0.08nM	-13.765	3GQI	Kd=33nM	-10.2
3JVF	Kd=292nM	-8.909	5HVG	Kd=0.63nM	-12.543
2Z58	Kd=16nM	-10.628	5IOH	Kd=778nM	-8.328
3OJM	Kd=130nM	-9.388	2VOG	Kd=210nM	-9.104
3U82	Kd=17.1nM	-10.589	2L0T	Kd=64uM	-5.717
2MFQ	Kd=5.2uM	-7.204	2DEN	Kd=17uM	-6.502
2ZA4	Kd=0.14nM	-13.434	3GTY	Kd=0.47nM	-12.717
2L2L	Kd=12.4nM	-10.779	6HER	Kd=40nM	-10.086
5U4K	Kd=15uM	-6.576	2BQZ	Kd=20uM	-6.406
2W80	Kd=4nM	-11.449	5VWY	Kd=1.3uM	-8.024
3KUD	Kd=1.7uM	-7.866	4AWX	Kd=0.5uM	-8.59
6HAR	Ki=61pM	-13.926	5ELU	Kd=414nM	-8.702
3ONA	Kd=0.68uM	-8.408	5OHP	Kd=3.5uM	-7.438

PDB entry	label	ΔG	PDB entry	label	ΔG
4JS0	Kd=5uM	-7.227	1NW9	Ki=0.013uM	-10.751
6UYU	Kd=2.0uM	-7.769	5F3X	Kd=3.11nM	-11.598
3WKT	Kd=0.1uM	-9.543	2F6A	Kd=0.2uM	-9.133
2LOX	Kd=190nM	-9.163	5AYR	Kd=1.959nM	-11.872
4KV5	Kd=1.7nM	-11.956	1KXV	Kd=25nM	-10.364
5DMJ	Kd=5.3nM	-11.282	6EJM	Kd=0.86nM	-12.359
5BRM	Kd=119nM	-9.44	4M1L	Kd=0.18uM	-9.195
6MBA	Kd=17nM	-10.592	2DJY	Kd=40uM	-5.996
2I3T	Kd=2.7uM	-7.592	6KM7	Kd=5.2uM	-7.204
1OZS	Kd=31uM	-6.147	2Q0O	Kd=14.9nM	-10.67
1LQS	Kd=4nM	-11.449	1GUA	Kd=40nM	-10.086
2M49	Kd=10uM	-6.816	5HK5	Kd=146nM	-9.319
4PQT	Kd=1.1uM	-8.123	5CYK	Kd=249nM	-9.003
1T5Z	Kd=33uM	-6.11	4ZRP	Kd=1.8nM	-11.922
5LZ3	Kd=19uM	-6.436	3CX8	Kd=340nM	-8.819
4K81	Kd=3.6uM	-7.421	5H7Y	Kd=125nM	-9.411
3DXD	Kd=0.34uM	-8.819	4XKH	Kd=0.94uM	-8.216
1G9I	Kd=0.12uM	-9.435	2DX5	Kd=0.33mM	-4.746
4JO6	Kd=1.53nM	-12.018	3SRI	Kd=165nM	-9.247
4S0S	Kd=11.4nM	-10.829	1T6B	Kd=0.4nM	-12.812
3IA3	Kd=93nM	-9.586	4LRZ	Kd=0.5uM	-8.59
2P47	Kd=116nM	-9.455	4GIQ	Kd=2.09uM	-7.743
3SKU	Kd=70nM	-9.754	6FBX	Kd=343nM	-8.813
3KZ1	Kd=110nM	-9.487	6ON9	Kd=10uM	-6.816
4KT3	Kd=0.26nM	-13.067	1EMV	Kd=24fM	-18.568
4HEM	Kd=10nM	-10.906	4DRA	Kd=1.17uM	-8.087
2KVQ	Kd=50uM	-5.864	1RKE	Kd=50nM	-9.953
4DJ9	Kd=99nM	-9.549	1WRD	Kd=409uM	-4.619
5G15	Kd=0.17uM	-9.229	1H0T	Kd=6uM	-7.119
6J4O	Kd=18nM	-10.558	6N85	Kd=0.24uM	-9.025
3ZWL	Kd=160nM	-9.265	2WVW	Kd=2uM	-7.769
5V5V	Kd=21nM	-10.467	5NQG	Kd=50nM	-9.953
4V0N	Kd=348nM	-8.805	2WG4	Kd=73.9nM	-9.722
5EQL	Kd=68nM	-9.771	4BXL	Kd=240nM	-9.025
2MJ5	Kd=4.3uM	-7.316	5V69	Kd=35.9nM	-10.15
5M72	Kd=33nM	-10.2	2LQH	Kd=85uM	-5.549
5Z7G	Kd=2.05uM	-7.755	6J9L	Kd=2115nM	-7.736
2P4A	Kd=180pM	-13.285	3TEI	Kd=0.5uM	-8.59
2SIC	Kd=71.2pM	-13.834	6DSL	Kd=500pM	-12.68
5VCB	Kd=62nM	-9.826	1Q69	Kd=900nM	-8.242
5HPY	Kd=0.13uM	-9.388	5MTN	Kd=23.5nM	-10.401
4X7E	Kd=7nM	-11.118	6GD5	Kd=3.8nM	-11.479
1X1W	Kd=26.4nM	-10.332	1P3Q	Kd=1.2uM	-8.072
2XGY	Kd=30uM	-6.166	6FUZ	Kd=3.19uM	-7.493
1P6A	Kd=35nM	-10.165	5SZH	Kd=5.2uM	-7.204
4GI3	Ki=0.68nM	-12.498	4B93	Kd=2.3uM	-7.687

PDB entry	label	ΔG	PDB entry	label	ΔG
5OEN	Kd=10nM	-10.906	5IMY	Kd=779nM	-8.328
3TU3	Kd=57nM	-9.876	2OMY	Kd=0.2uM	-9.133
3RZW	Kd=68nM	-9.771	3E1Z	Ki=0.036nM	-14.238
5O2U	Kd=0.69nM	-12.489	2G2W	Kd=582nM	-8.5
2P8Q	Kd=15.6nM	-10.643	6GBH	Kd=69nM	-9.763
1VYH	Kd=0.08uM	-9.675	5JW7	Kd=9.09uM	-6.873
4G01	Kd=3.4uM	-7.455	3H2V	Kd=22.1uM	-6.347
4PC0	Kd=0.2uM	-9.133	5U4M	Kd=0.39nM	-12.827
4YH7	Kd=0.15uM	-9.303	1OGY	Kd=0.5nM	-12.68
2KWK	Kd=2.3uM	-7.687	1ZVY	Kd=0.1nM	-13.633
3FPU	Kd=0.12nM	-13.525	4DZV	Ki=11nM	-10.85
4YEB	Kd=17nM	-10.592	6GTQ	Kd=367nM	-8.773
2Y8T	Kd=14.2nM	-10.699	3VV2	Kd=52pM	-14.02
6J7W	Kd=0.3nM	-12.983	2K8B	Kd=1.8mM	-3.742
2WEL	Kd=0.3uM	-8.893	1Y6K	Kd=1nM	-12.27
2RU4	Kd=126nM	-9.406	2WP3	Kd=0.794uM	-8.316
6ER6	Kd=51nM	-9.942	6MAV	Ki=33.5pM	-14.281
1H59	Kd=37nM	-10.132	2HWN	Kd=2nM	-11.859
4MP0	Kd=8.7nM	-10.989	4KFZ	Kd=94nM	-9.58
6JJW	Kd=8.2nM	-11.024	1WA7	Kd=9.58uM	-6.842
4W6Y	Kd=1.58nM	-11.999	4LAD	Kd=38nM	-10.116
6F2G	Kd=3.9nM	-11.464	3REA	Kd=179nM	-9.198
3QHY	Kd=1.1pM	-16.303	1JTD	Ki=27.2pM	-14.404
3BS5	Kd=92.5nM	-9.589	2KA4	Kd=58nM	-9.866
1R8U	Kd=13nM	-10.751	3UL1	Kd=2.7nM	-11.682
2LVO	Kd=12.8uM	-6.67	3CX7	Kd=520nM	-8.567
1L8C	Kd=7nM	-11.118	5ZQV	Kd=0.26uM	-8.977
2ROZ	Kd=0.32uM	-8.854	2BWE	Kd=80uM	-5.585
1ES0	IC50=0.08uM	-9.675	4ASW	Kd=100nM	-9.543
4Z9V	Kd=12uM	-6.709	3IDB	Kd=11.3nM	-10.834
1I8L	Kd=0.2uM	-9.133	5XQZ	Kd=12.3uM	-6.694
1PXV	Ki=0.31nM	-12.963	1Y3C	Ki=0.025nM	-14.454
2LY4	Kd=346nM	-8.808	6D68	IC50=250nM	-9.001
3QLU	Kd=11nM	-10.85	5ZAU	Kd=60nM	-9.846
2FJU	Kd=5.3uM	-7.192	1B2S	Kd=157pM	-13.366
1GL0	Ki=0.8nM	-12.402	2V9T	Kd=8.2nM	-11.024
5LHN	Kd=11.2nM	-10.839	1U7F	Kd=58nM	-9.866
3U9D	Kd=0.5uM	-8.59	2WJV	Kd=0.2uM	-9.133
1PSB	Kd=20uM	-6.406	5MK0	Kd=3.1uM	-7.51
1FFW	Kd=1.2uM	-8.072	1Y34	Ki=0.52nM	-12.657
6H6Y	Kd=0.163nM	-13.344	4IOS	Kd=19nM	-10.526
3ME2	Kd=68pM	-13.861	4ONS	Kd=15.8nM	-10.636
4C4P	Kd=0.25uM	-9.001	3C5T	IC50=0.6nM	-12.572
5SV3	Kd=0.627nM	-12.546	6F9S	Kd=1.62uM	-7.894
2J12	Kd=20nM	-10.496	3GXU	Kd=203nM	-9.124
5IJ0	Kd=7.9uM	-6.956	2M5A	Kd=48nM	-9.978

PDB entry	label	ΔG	PDB entry	label	ΔG
1EER	Kd=1nM	-12.27	1RPQ	IC50=0.032uM	-10.218
4K0A	Kd=0.18nM	-13.285	3DVM	Kd=4.32nM	-11.403
2MUR	Kd=9.26uM	-6.862	6UMT	Kd=2.6nM	-11.704
1GPW	Kd=0.019uM	-10.526	2WG3	Kd=73.6nM	-9.725
1ZJD	IC50=1.28nM	-12.124	3LBX	Kd=400nM	-8.722
2RR3	Kd=2.1uM	-7.741	6GVL	Kd=44uM	-5.939
4YN0	Kd=85nM	-9.639	1YVB	Ki=6.5nM	-11.161
1OP9	Kd=0.7nM	-12.481	3HH2	Kd=12.3nM	-10.784
3D7T	Kd=70uM	-5.664	5L21	Kd=3.7nM	-11.495
6S8S	Kd=410nM	-8.708	5TZP	Kd=30nM	-10.256
4ZGM	IC50=0.38nM	-12.843	2N8J	Kd=10.7nM	-10.866
4K5B	Kd=643pM	-12.531	1XDT	Kd=10nM	-10.906
2H4M	Kd=42nM	-10.057	4KGQ	Kd=36nM	-10.148
3MC0	Kd=0.125uM	-9.411	3HG0	Kd=16nM	-10.628
3IXE	IC50=2.3uM	-7.687	2OUL	Ki=1.7nM	-11.956
1B3S	Kd=117pM	-13.54	2KQS	Kd=55.3uM	-5.804
2RNR	Kd=150nM	-9.303	1WR6	Kd=231uM	-4.957
5NVL	Kd=0.3nM	-12.983	1NYU	Kd=10nM	-10.906
3ZYJ	Kd=7.9nM	-11.046	4EEE	Kd=0.2nM	-13.223
2MWS	Kd=45uM	-5.926	6F0G	Kd=0.055uM	-9.897
3ZU7	Kd=6.6nM	-11.152	3O5T	Kd=1.5uM	-7.94
4CU4	Kd=1.2uM	-8.072	2QJA	Kd=1.1nM	-12.213
2OMU	Kd=0.6nM	-12.572	1UUG	Kd=1.3uM	-8.024
6FG8	Kd=0.98uM	-8.192	5OMB	Kd=0.43uM	-8.679
4REY	Kd=108nM	-9.498	4XXW	Kd=5.12uM	-7.213
6MC9	Kd=305nM	-8.883	1LP1	Kd=2uM	-7.769
3BK3	Kd=22nM	-10.44	5E6P	Kd=2uM	-7.769
5WUJ	Kd=422nM	-8.691	4G6U	Kd=17.8nM	-10.565
3O43	Ki=9nM	-10.969	4H2W	Kd=1.73uM	-7.855
5MA4	Kd=52pM	-14.02	2VER	Kd=13.1uM	-6.657
5MN2	Kd=2.6uM	-7.614	1US7	Kd=1.8uM	-7.832
3MHP	Kd=2.75uM	-7.581	4DOQ	Ki=140nM	-9.344
5J57	Kd=0.02nM	-14.586	5YWR	Kd=39nM	-10.101
5KVE	Kd=35nM	-10.165	2LWW	Kd=57nM	-9.876
5VZ4	Kd=8nM	-11.039	2NOJ	Kd=13nM	-10.751
6M7L	Kd=10.4uM	-6.793	4D2G	Kd=5.56uM	-7.164
3BH6	Kd=95nM	-9.573	5D1M	Kd=62nM	-9.826
4YYP	Kd=280nM	-8.933	3UZ0	Kd=0.56uM	-8.523
1JSP	Kd=50uM	-5.864	5EB1	Kd=140nM	-9.344
3NCB	Kd=93mM	-1.406	2XA0	Kd=35.3nM	-10.16
2P43	Kd=23nM	-10.413	4GEH	Kd=11.7nM	-10.813
5TWA	Kd=66nM	-9.789	3L89	Kd=284nM	-8.925
5C67	Ki=89pM	-13.702	6IW8	Kd=332nM	-8.833
5M36	Kd=2.75uM	-7.581	4BRU	Kd=200nM	-9.133
5W8T	Kd=27.06uM	-6.227	3GC3	Kd=2.1uM	-7.741
1TY4	Kd=6.4nM	-11.171	6MUD	Kd=1.02uM	-8.168

PDB entry	label	ΔG	PDB entry	label	ΔG
1EAW	Kd=49.7pM	-14.047	4X4M	Kd=8.8nM	-10.982
1WVE	Kd=7nM	-11.118	1OYV	Ki=9nM	-10.969
5O2S	Kd=3.9nM	-11.464	1EES	Kd=20nM	-10.496
4EOZ	Kd=1uM	-8.18	3U8X	Kd=3.4uM	-7.455
5UN5	Kd=18nM	-10.558	3L9J	Kd=0.34nM	-12.908
2C7N	Kd=6.4uM	-7.081	4G6V	Kd=21.1nM	-10.464
2PKG	Kd=1.06uM	-8.145	2XHE	Kd=3.9nM	-11.464
4AFZ	Kd=0.9nM	-12.332	6H6Z	Kd=4.58nM	-11.369
5LI1	Kd=0.47uM	-8.627	5AGW	IC50=10.2nM	-10.895
2B10	Kd=10uM	-6.816	1TZN	Kd=170pM	-13.319
5NZZ	Kd=1.7uM	-7.866	3K2M	Kd=7nM	-11.118
5Y1Z	Kd=4.3uM	-7.316	4AQE	Kd=2.4uM	-7.661
4EIZ	Kd=3nM	-11.619	4CDK	Kd=1.9uM	-7.8
4LGP	Kd=0.23nM	-13.14	4AOQ	Kd=201.8nM	-9.127
1RKC	Kd=39nM	-10.101	1XQH	Kd=0.2uM	-9.133
6I3F	Kd=20.3nM	-10.487	1XT9	Kd=200nM	-9.133
4KT5	Kd=1.1uM	-8.123	2LPB	Kd=10.1uM	-6.811
6HUL	Kd=0.3uM	-8.893	5TVQ	Kd=0.88uM	-8.255
1XD3	Kd=0.3uM	-8.893	3S8V	Kd=50nM	-9.953
2XQW	Kd=0.18uM	-9.195	5NVN	Kd=55nM	-9.897
3BEG	Kd=50nM	-9.953	3G3A	Kd=0.43uM	-8.679
3EIQ	Kd=110nM	-9.487	6OAO	IC50=1.63nM	-11.98
2POP	Kd=4.2uM	-7.33	1PJM	Kd=180nM	-9.195
2EFD	Kd=7.3nM	-11.093	6DGF	IC50=9.5nM	-10.937
2MRO	Kd=150uM	-5.213	3TZ1	Kd=0.141uM	-9.34
2GOX	Kd=0.8nM	-12.402	5OMN	Kd=9.5nM	-10.937
1WA8	Kd=11nM	-10.85	3QQ8	Kd=1.5uM	-7.94
1WLP	Kd=0.64uM	-8.444	2A7U	Kd=120nM	-9.435
3EGG	Kd=8.7nM	-10.989	6NE1	Kd=1.4nM	-12.07
4CJ1	Kd=48nM	-9.978	4H3P	Kd=2uM	-7.769
4DS8	Kd=7.7uM	-6.971	1AZZ	Ki=510pM	-12.668
2CPK	Ki=2.3nM	-11.777	3QBV	Kd=478nM	-8.617
5MR5	Kd=80nM	-9.675	4IKA	Kd=57.5uM	-5.781
3CRK	Kd=8.3uM	-6.927	5HYC	Kd=15.2uM	-6.569
1EZS	Ki=0.18nM	-13.285	3Q9N	Kd=17nM	-10.592
2LVP	Kd=13.4uM	-6.643	5OC7	Kd=33nM	-10.2
4QXA	Kd=0.32uM	-8.854	2QJ9	Kd=2.4nM	-11.751
6KBM	Kd=0.60uM	-8.482	3GJ3	Kd=49uM	-5.876
5LXQ	Kd=0.64uM	-8.444	2A78	Kd=60nM	-9.846
2L14	Kd=3.1uM	-7.51	1UEL	Kd=3.4uM	-7.455
1Y4A	Ki=1.2nM	-12.162	2NZ1	Ki=18nM	-10.558
3OKY	Kd=1.3uM	-8.024	2B87	Kd=100nM	-9.543
5MY6	Kd=3.99nM	-11.45	4IU3	Kd=20.83nM	-10.472
5H9B	Kd=674.1nM	-8.413	2V52	Kd=1.9uM	-7.8
5FT8	Kd=117nM	-9.45	3UZE	Kd=8nM	-11.039
1P9D	Kd=12.9uM	-6.666	3WQB	Kd=1.8nM	-11.922

PDB entry	label	ΔG	PDB entry	label	ΔG
6ASR	Kd=14.9uM	-6.58	1ZSG	Kd=7.5uM	-6.987
6BX4	Kd=210nM	-9.104	3ZEU	Kd=0.29uM	-8.913
4AG2	Kd=6.2nM	-11.189	2M0J	Kd=12nM	-10.798
3R85	Kd=41.3uM	-5.977	1L4Z	Kd=196.6nM	-9.143
6P7Y	Kd=0.56nM	-12.613	3QC8	Kd=11.2uM	-6.749
5JEM	Kd=6.02uM	-7.117	2KTF	Kd=23uM	-6.323
1TA3	Ki=9nM	-10.969	4U4C	Kd=310nM	-8.873
5EJC	Kd=197nM	-9.142	3NVN	Kd=9.4nM	-10.943
6BA6	Kd=162uM	-5.168	5XNP	Kd=281.8nM	-8.93
2CJS	Kd=0.1uM	-9.543	1XJ7	Kd=39uM	-6.011
2AQ2	Kd=11nM	-10.85	4UYP	Kd=20pM	-14.586
4HGK	Kd=0.19nM	-13.253	2XPX	Kd=150nM	-9.303
4J2Y	Ki=0.88nM	-12.345	3TWI	Kd=0.6uM	-8.482
3GJ5	Kd=47uM	-5.9	4XWJ	Kd=4.45nM	-11.386
1UL1	Kd=60nM	-9.846	2HQW	Kd=2nM	-11.859
1KAC	Kd=14.8nM	-10.674	4GLV	Kd=2.92nM	-11.635
4ZII	IC50=66nM	-9.789	2GNG	IC50=6uM	-7.119
5D3I	Kd=0.6nM	-12.572	2AQ1	Kd=5.5nM	-11.26
5J8H	Kd=153nM	-9.291	2P42	Kd=23nM	-10.413
2KC8	Kd=200nM	-9.133	2I25	Kd=1.0nM	-12.27
4D0N	Kd=18.1uM	-6.465	4GH7	Kd=5.77nM	-11.232
4X7F	Kd=28nM	-10.297	6Q69	Kd=0.17uM	-9.229
5L8J	Kd=2uM	-7.769	4OVN	Kd=46nM	-10.003
3OUN	Kd=58nM	-9.866	2RVB	Kd=143.3nM	-9.33
1LDT	Ki=1.8nM	-11.922	3CH5	Kd=120uM	-5.345
2IFG	Kd=0.9nM	-12.332	2W84	Kd=0.07uM	-9.754
1KTZ	Kd=290nM	-8.913	4AYI	Kd=2nM	-11.859
5A6W	Kd=31nM	-10.237	5CX3	Kd=0.54uM	-8.545
2YJ1	IC50=26nM	-10.341	4KDI	Kd=0.71uM	-8.383
6NE4	Kd=1.6nM	-11.991	4AUO	Kd=0.4uM	-8.722
1XX9	IC50=3nM	-11.619	4CJ2	Kd=11nM	-10.85
4MJS	Kd=21.6nM	-10.45	6AAF	Kd=161nM	-9.261
4WND	Kd=32.9nM	-10.201	6GVK	Kd=2.0uM	-7.769
2W83	Kd=0.42uM	-8.693	5X0W	Kd=3.7uM	-7.405
3T0Y	Kd=193nM	-9.154	1ZV5	Kd=10nM	-10.906
3VPJ	Kd=2.77nM	-11.666	6O8B	Kd=11uM	-6.76
2C1M	Kd=1.1nM	-12.213	2PHE	Kd=15uM	-6.576
2Y9M	Kd=2nM	-11.859	3D7U	Kd=30uM	-6.166
2JTI	Kd=0.588uM	-8.494	5JW9	Kd=0.086uM	-9.632
6IB8	Kd=94nM	-9.58	3UL0	Kd=2.3nM	-11.777
4L67	Kd=0.32uM	-8.854	4RE1	Kd=1.2uM	-8.072
2A9H	Kd=900nM	-8.242	4NZW	Kd=504nM	-8.585
2PTT	Kd=4uM	-7.359	5ME5	Kd=2.15uM	-7.727
4I6L	Kd=20nM	-10.496	6FP7	Kd=3nM	-11.619
1Z7X	Kd=0.29fM	-21.182	2JTT	Kd=4.9uM	-7.239
2XTT	Ki=20pM	-14.586	4N7V	Kd=32nM	-10.218

PDB entry	label	ΔG	PDB entry	label	ΔG
2B0Z	Kd=100uM	-5.453	5JSB	Kd=0.15uM	-9.303
4PL8	Kd=0.32uM	-8.854	3B08	Kd=17.2uM	-6.495
3IFQ	Kd=116nM	-9.455	6B12	Kd=8.1nM	-11.031
2VOH	Kd=1nM	-12.27	2XBB	Kd=11uM	-6.76
2Z8V	Kd=4.8nM	-11.341	5D50	Kd=2.6uM	-7.614
2HRK	Kd=9nM	-10.969	3WA5	Kd=28.1nM	-10.295
2KWU	Kd=51uM	-5.852	1KXT	Kd=235nM	-9.037
4HX3	Ki=6nM	-11.209	1Y3B	Ki=0.31nM	-12.963
2V51	Kd=1.0uM	-8.18	3K75	Kd=110nM	-9.487
6J9M	Kd=81nM	-9.668	3D5R	Kd=19nM	-10.526
2MZD	Kd=0.877uM	-8.258	2OOB	Kd=57uM	-5.786
4Y5O	Kd=1.4uM	-7.981	1YRT	Kd=1nM	-12.27
1UAD	Kd=137nM	-9.357	4K5A	Kd=10.3nM	-10.889
5F4E	Kd=48nM	-9.978	2M04	Kd=10nM	-10.906
2LQC	Kd=2.9uM	-7.549	4FZV	Kd=13.3nM	-10.738
4JEU	Kd=1.4nM	-12.07	1TM5	Ki=0.017nM	-14.682
3ZKQ	Kd=1.2nM	-12.162	5OAQ	Kd=58nM	-9.866
2B7C	Kd=0.4uM	-8.722	2VDA	Kd=3uM	-7.529
2AJF	Kd=10nM	-10.906	1TM1	Ki=0.003nM	-15.709
3FF7	Kd=200uM	-5.043	2MBB	Kd=130uM	-5.298
5XDA	Kd=1.6uM	-7.902	4MRT	Kd=0.92uM	-8.229
2HD5	Kd=2.8uM	-7.57	2G81	Ki=13.6nM	-10.724
2OMX	Kd=120nM	-9.435	5WRV	Kd=0.73uM	-8.366
1HCF	Kd=260pM	-13.067	2DSP	Kd=3uM	-7.529
3OJ2	Kd=130nM	-9.388	2K3S	Kd=2.7uM	-7.592
6OQQ	Kd=28nM	-10.297	4I9X	Kd=5.96nM	-11.213
3P71	Kd=0.38uM	-8.753	5UFQ	Kd=6.4nM	-11.171
5YR0	Kd=7.6uM	-6.979	1WPX	Ki=1.80nM	-11.922
3QHT	Kd=82nM	-9.661	5V5I	Kd=463nM	-8.636
1K93	Kd=20nM	-10.496	1TBA	Kd=1nM	-12.27
5JMC	Kd=86nM	-9.632	4YC7	Kd=1.89uM	-7.803
3FHC	Kd=21.6nM	-10.45	6GBU	Kd=248nM	-9.005
1WDW	Kd=14.3nM	-10.695	5B64	Kd=70nM	-9.754
5SXP	Kd=1.59uM	-7.905	1ZC4	Kd=42nM	-10.057
2ABZ	Ki=2.8nM	-11.66	5EOA	Kd=0.1uM	-9.543
5MAD	Kd=1.1nM	-12.213	5M2I	Kd=0.54nM	-12.635
4UI0	Kd=88nM	-9.619	2XZE	Kd=113nM	-9.471
6M9K	Kd=12uM	-6.709	3HCT	Kd=1.48uM	-7.948
1YRU	Kd=1nM	-12.27	4YVQ	Kd=2.03uM	-7.761
1OEY	Kd=4nM	-11.449	2RUK	Kd=24.21uM	-6.293
2VLP	Kd=0.543pM	-16.721	4ZK9	Kd=0.24nM	-13.115
1TO1	Ki=0.46nM	-12.729	1DJS	Kd=41nM	-10.071
4OYD	Kd=220pM	-13.166	2L9S	Kd=2.2uM	-7.713
5MA6	Kd=303pM	-12.977	2B11	Kd=5uM	-7.227
3SJH	Kd=3.4uM	-7.455	5NVM	Kd=14nM	-10.707
5AJK	Kd=2640nM	-7.605	2ODG	Kd=0.59uM	-8.492

PDB entry	label	ΔG	PDB entry	label	ΔG
2JY6	Kd=20uM	-6.406	2OMV	Kd=1.2nM	-12.162
4GN4	Kd=0.253uM	-8.994	4BSR	Kd=2nM	-11.859
4YJ4	Kd=9.9nM	-10.912	2QXV	Kd=0.38uM	-8.753
5ET1	Kd=107nM	-9.503	4LGR	Kd=0.11nM	-13.577
5D28	Kd=27nM	-10.318	2L29	Kd=15.3nM	-10.655
4HCN	Kd=106.7uM	-5.415	2WWX	Kd=79nM	-9.683
1F34	Kd=0.1nM	-13.633	6F3Z	Kd=405nM	-8.715
3DDC	Kd=0.77uM	-8.335	6AW2	Kd=57nM	-9.876
2P49	Kd=23nM	-10.413	6G10	Kd=5.9nM	-11.219
4ETW	Kd=3.1uM	-7.51	1ZVH	Kd=70nM	-9.754
6IWD	Kd=18.2nM	-10.552	2L0F	Kd=172uM	-5.132
5XLN	Kd=2.48uM	-7.642	4ILW	Ki=5.8nM	-11.229
4MIT	Kd=0.178uM	-9.202	3K6G	Kd=16.5nM	-10.61
1EFN	Kd=0.38uM	-8.753	5WGG	Kd=0.7uM	-8.391
5NL1	Kd=174nM	-9.215	2HSQ	Kd=6.61nM	-11.152
1LFD	Kd=1.9uM	-7.8	3KW5	Kd=385nM	-8.745
1E4K	Kd=1.7uM	-7.866	3OSL	Kd=1.8nM	-11.922
4JW2	Kd=3.7nM	-11.495	2J0T	Ki=0.4nM	-12.812
4AP2	Kd=20nM	-10.496	5KHO	Kd=0.9uM	-8.242
3A4S	Kd=2.81uM	-7.568	2Z3R	Kd=38pM	-14.206
3GS2	Kd=9.2nM	-10.956	3LMS	Ki=1.2nM	-12.162
3T6G	Kd=30nM	-10.256	5LXM	Kd=0.18uM	-9.195
2OMT	Kd=100nM	-9.543	1VRK	Kd=6.8nM	-11.135
3NCC	Kd=212mM	-0.918	3VEP	Kd=10.3uM	-6.799
1ATN	Kd=0.45nM	-12.742	6IU7	Kd=0.9nM	-12.332
1KXQ	Kd=3.5nM	-11.528	1MCV	Ki=98nM	-9.555
5HGG	Kd=0.054nM	-13.998	4N7Z	Kd=177nM	-9.205
5NQW	Kd=1.4nM	-12.07	3GJ4	Kd=6.6uM	-7.063
5JJD	Kd=9.2uM	-6.866	2W81	Kd=4nM	-11.449
2P1L	Kd=2.3uM	-7.687	1Q0W	Kd=277uM	-4.85
5OY9	Kd=3.75uM	-7.397	4HEP	Kd=300nM	-8.893
6ARQ	Kd=10.2uM	-6.805	4MI8	Kd=1.38uM	-7.989
1X75	Kd=17.5nM	-10.575	5JG6	Kd=1.6uM	-7.902
5K22	Kd=25nM	-10.364	6MKZ	Kd=0.2nM	-13.223
3WKN	Kd=1.6nM	-11.991	2A24	Kd=30uM	-6.166
3RBB	Kd=12.3nM	-10.784	2X2D	Kd=6.1uM	-7.109
3W8I	Kd=2.15nM	-11.816	5TGH	Kd=0.95uM	-8.21
2I3S	Kd=3.6uM	-7.421	5UFE	Kd=50.3nM	-9.95
4AFQ	Kd=2.4nM	-11.751	3M0A	Kd=1.7uM	-7.866
4DT1	Kd=0.56uM	-8.523	4CMM	Kd=0.8uM	-8.312
6AZP	Kd=35.1nM	-10.163	4BQD	Kd=0.5nM	-12.68
4DM6	Kd=1.5uM	-7.94	2KT5	Kd=28uM	-6.207
3EAB	Kd=12uM	-6.709	5K8Q	Kd=0.9nM	-12.332
6NDZ	Kd=0.2nM	-13.223	3DA7	Kd=0.13uM	-9.388
5DJU	Kd=11.4nM	-10.829	1FMO	Ki=2.3nM	-11.777
5EO9	Kd=0.37uM	-8.768	6FC3	Kd=20nM	-10.496

PDB entry	label	ΔG	PDB entry	label	ΔG
1ZLI	Ki=1.3nM	-12.114	5WPA	Kd=0.55uM	-8.534
6FUB	Kd=29nM	-10.276	3S2K	Kd=71.5nM	-9.742
5SWK	Kd=2.57uM	-7.621	3MZG	Kd=4.97mM	-3.141
3GJ6	Kd=6.5uM	-7.072	4B1X	Kd=4.34uM	-7.311
2K2U	Kd=360nM	-8.785	6I07	Kd=10nM	-10.906
1U0S	Kd=0.23uM	-9.05	6H15	Kd=12.8nM	-10.76
4PVZ	Kd=27.3nM	-10.312	1ZR0	Ki=13nM	-10.751
5F5O	Kd=162nM	-9.257	2P46	Kd=116nM	-9.455
1FC2	Kd=22.5nM	-10.426	2K79	Kd=0.67mM	-4.327
5OVW	Ki=0.94nM	-12.306	1G5J	Kd=0.6nM	-12.572
4CT0	Kd=28nM	-10.297	5YQZ	IC50=8.1nM	-11.031
2X1W	Kd=16nM	-10.628	3O34	Kd=8.8uM	-6.892
5CFF	Kd=1.07uM	-8.14	6BX5	Kd=14nM	-10.707
6CO2	Kd=1.2uM	-8.072	5D1K	Kd=180nM	-9.195
1VYT	Kd=20nM	-10.496	4BPX	Kd=0.25uM	-9.001
2ES4	Kd=5nM	-11.317	6N9D	Ki=17.8pM	-14.655
3WO3	Kd=69nM	-9.763	3UX9	Kd=0.747nM	-12.442
4JRA	Kd=28nM	-10.297	1L2W	Kd=0.3nM	-12.983
1JWH	Kd=12.6nM	-10.77	2VE7	Kd=4uM	-7.359
5WOS	Kd=353nM	-8.796	2V8S	Kd=22uM	-6.35
5DC4	Kd=35nM	-10.165	3ULA	Kd=2.84nM	-11.652
3GNI	Kd=12nM	-10.798	6DCN	Kd=1.4uM	-7.981
2G45	Kd=2.82uM	-7.566	4UYQ	Kd=14pM	-14.797
2Z8W	Kd=4.7nM	-11.353	1Y33	Ki=1.7nM	-11.956
4NOO	Kd=4.92nM	-11.326	1U7V	Kd=296nM	-8.901
4HNJ	Kd=3nM	-11.619	2V6X	Kd=28uM	-6.207
4KT1	Kd=56.5nM	-9.881	3V3K	Kd=38.7nM	-10.105
3ZYI	Kd=7.3nM	-11.093	4XL5	Kd=1.4nM	-12.07
1GLA	Kd=0.18uM	-9.195	3LAQ	Kd=0.17nM	-13.319
3Q9U	Kd=5.2nM	-11.294	3F7P	Kd=49uM	-5.876
4F48	Kd=3.2uM	-7.491	5F5S	Kd=22nM	-10.44
5FW5	Kd=80nM	-9.675	6KBR	Kd=8.91pM	-15.065
6E3J	Kd=13.98nM	-10.708	5JSN	Kd=0.8uM	-8.312
2N01	Kd=1uM	-8.18	2K2S	Kd=53nM	-9.919
3KUC	Kd=0.442uM	-8.663	4IHL	Kd=346nM	-8.808
3N06	Kd=3.1mM	-3.42	4KA2	Kd=8.4pM	-15.1
2K5B	Kd=4uM	-7.359	2MNU	Kd=230nM	-9.05
2FTL	Kd=60fM	-18.025	6N6R	Kd=3.8uM	-7.389
6SAZ	Ki=0.14nM	-13.434	5V62	IC50=25nM	-10.364
3QI2	Kd=45nM	-10.016	2LVQ	Kd=22.6uM	-6.334
3KNB	Kd=0.94uM	-8.216	3GB8	Kd=6.5uM	-7.072
3AJB	Kd=40.8nM	-10.074	4P6I	Kd=290nM	-8.913
2FYL	Kd=2.8uM	-7.57	2KXW	Kd=10nM	-10.906
4GN5	Kd=31.1nM	-10.235	1YK0	Kd=0.96nM	-12.294
3G9W	Kd=36uM	-6.058	4R8I	Kd=1.4nM	-12.07
2VYR	Kd=44nM	-10.029	3ONL	Kd=12uM	-6.709

PDB entry	label	ΔG	PDB entry	label	ΔG
5XOD	Kd=28.1nM	-10.295	3WRX	Kd=2.1uM	-7.741
3O6B	Kd=227nM	-9.058	2ARP	Kd=430nM	-8.679
1USU	Kd=3.3uM	-7.473	1DU3	Kd=15.9nM	-10.632
6QB3	Kd=2.38uM	-7.666	6KBN	Kd=1.46uM	-7.956
6THG	Kd=4nM	-11.449	5B76	Kd=5.8uM	-7.139
4BFI	Kd=0.6uM	-8.482	4R8G	Kd=0.49uM	-8.602
3BRT	IC50=36nM	-10.148	4C5G	Kd=1.03uM	-8.162
4UF1	Kd=6.98uM	-7.029	2BE6	Kd=2.63nM	-11.697
5IMM	Kd=3.5nM	-11.528	3U9Z	Kd=1.54uM	-7.924
4MQV	Kd=2.9uM	-7.549	2VLQ	Kd=22.8pM	-14.508
4D0G	Kd=1.8uM	-7.832	2LUH	Kd=0.7uM	-8.391
2G2U	Kd=1.25uM	-8.048	5DJT	Kd=17nM	-10.592
1GNG	Kd=39nM	-10.101	5UK5	Kd=0.81uM	-8.305
2JKI	Kd=1.64uM	-7.887	4BRW	Kd=50nM	-9.953
3EJH	Kd=5uM	-7.227	5XIU	Kd=25uM	-6.274
6B8N	Kd=210nM	-9.104	3RT0	Kd=1.2uM	-8.072
3H6S	Ki=0.08nM	-13.765	4ZGY	Kd=0.7uM	-8.391
5O0W	Kd=73.83pM	-13.813	1PK1	Kd=54nM	-9.908
4DID	Kd=6uM	-7.119	4OL0	Kd=4.7nM	-11.353
3UKX	Kd=0.03nM	-14.346	6OQK	Kd=42nM	-10.057
5SZK	Kd=1.1uM	-8.123	1LZW	Kd=0.33uM	-8.836
1O9A	Kd=1uM	-8.18	2VOI	Kd=1.1nM	-12.213
5XCO	Kd=8.9nM	-10.975	6AKM	Kd=6.9uM	-7.036
3WWN	Kd=6.62uM	-7.061	5JLV	Kd=15nM	-10.666
4FQ0	Kd=0.87uM	-8.262	4QLP	Kd=0.23nM	-13.14
5GWO	Kd=30nM	-10.256	1RJC	Kd=77pM	-13.788
3SUA	Kd=18.9uM	-6.44	1XG2	Kd=5nM	-11.317
3LB8	Kd=66uM	-5.699	6B05	Kd=1.9uM	-7.8
2BYM	Kd=2.3uM	-7.687	4ETP	Kd=0.37uM	-8.768
1C9T	Ki=1.0nM	-12.27	4HGM	Kd=16nM	-10.628
1U0I	Kd=70nM	-9.754	5FR1	Kd=3.3uM	-7.473
1SG1	Kd=186nM	-9.176	6MOE	Kd=1.89nM	-11.893
5JYL	Kd=8.7nM	-10.989	5U4Y	Kd=25nM	-10.364
1PD7	Kd=0.3uM	-8.893	6IN9	Kd=1.2uM	-8.072
5J56	Kd=3.2nM	-11.581	5IP4	Kd=451nM	-8.651
4KR0	Kd=16.7nM	-10.603	1TH1	Kd=2.1nM	-11.83
1AY7	Kd=1uM	-8.18	1CMX	Ki=1.7uM	-7.866
2NQD	Ki=39pM	-14.191	4YDH	Kd=2.55uM	-7.626
6A7V	Kd=0.6nM	-12.572	2N2H	Kd=0.93uM	-8.223
3V96	Ki=1.1nM	-12.213	4XXB	Kd=1.48uM	-7.948
1C9P	Ki=1.0nM	-12.27	3M18	Kd=180pM	-13.285
4DX8	Kd=1.24uM	-8.052	5CZF	Kd=100pM	-13.633
5N47	Kd=7nM	-11.118	6PNP	Kd=299.2nM	-8.894
3P95	Ki=1.5uM	-7.94	2JZN	Kd=0.5mM	-4.5
5MR4	Kd=220nM	-9.076	5TZQ	Kd=16nM	-10.628
5KY9	Kd=5.9uM	-7.129	5VU0	Kd=2.5nM	-11.727

PDB entry	label	ΔG	PDB entry	label	ΔG
4P3Y	Kd=146nM	-9.319	1UUZ	Ki=25nM	-10.364
3EBA	Kd=2.36nM	-11.761	5VMO	Kd=887nM	-8.251
1AVZ	Kd=15.8uM	-6.546	1LW6	Kd=2pM	-15.949
5MV8	Kd=1.4uM	-7.981	1WR1	Kd=14.8uM	-6.584
3IOL	IC50=500nM	-8.59	6S29	Kd=7.75nM	-11.057
3E2U	Kd=2.3uM	-7.687	2XB6	Kd=115nM	-9.46
3KYI	Kd=218uM	-4.992	2V5Q	Kd=71nM	-9.746
5H3J	Kd=0.27uM	-8.955	1TM4	Ki=0.13nM	-13.478
2IHS	Kd=40nM	-10.086	6ATK	Kd=0.43uM	-8.679
2KBW	Kd=0.32uM	-8.854	3OIQ	Kd=3.8uM	-7.389
1Y1K	Ki=0.3nM	-12.983	5Z78	Kd=0.9uM	-8.242
2HLE	Kd=40nM	-10.086	5H07	Kd=2.82uM	-7.566
2P45	Kd=116nM	-9.455	5WB8	Kd=2.8uM	-7.57
1LX5	Kd=1.2nM	-12.162	1RLB	Kd=0.8uM	-8.312
4MSM	Kd=3uM	-7.529	1I5K	Kd=42nM	-10.057
3G3B	Kd=34nM	-10.182	3IT8	Kd=43pM	-14.133
5B78	Kd=1.88uM	-7.806	2KHS	Kd=0.36uM	-8.785
5WY2	Kd=0.54uM	-8.545	3TL8	Kd=3.5uM	-7.438
1Q68	Kd=400nM	-8.722	3QS7	Kd=40nM	-10.086
2WD5	Kd=22nM	-10.44	4P1N	Kd=0.36uM	-8.785
2AW2	Kd=25nM	-10.364	5UWC	Kd=17.8nM	-10.565
2L1L	Kd=7nM	-11.118	1I4D	Kd=3uM	-7.529
4I0C	Kd=460nM	-8.64	5ZVM	IC50=0.21uM	-9.104
3OGO	Kd=0.32nM	-12.944	2BP7	Kd=1nM	-12.27
1E3U	Kd=1uM	-8.18	2KXH	Kd=14uM	-6.617
6GJQ	Kd=25nM	-10.364	6CWG	Kd=0.102nM	-13.621
4RWS	IC50=6nM	-11.209	1XXD	IC50=600nM	-8.482
1VG0	Kd=5nM	-11.317	1A22	Kd=0.34nM	-12.908
6IM9	Kd=25nM	-10.364	1Q5W	Kd=126uM	-5.316
4W6X	Kd=5.25nM	-11.288	1Z0K	Kd=7.7uM	-6.971
2MRE	Kd=19uM	-6.436	4PLO	Kd=0.038uM	-10.116
5C4V	Kd=1.1uM	-8.123	3KZ0	Ki=273nM	-8.948
1QA9	Kd=1uM	-8.18	1SHY	Kd=90nM	-9.605
1SHZ	Kd=3uM	-7.529	6FGO	Kd=440nM	-8.666
2OIN	Kd=0.51uM	-8.578	1AK4	Kd=17uM	-6.502
3QBQ	Kd=230nM	-9.05	2OT3	Kd=1.8uM	-7.832
6P7S	Kd=0.24nM	-13.115	3M9D	Kd=3.4uM	-7.455
4NQW	Kd=0.42uM	-8.693	4KRL	Kd=47nM	-9.99
3ZNZ	Kd=196nM	-9.145	3KV4	Kd=1uM	-8.18
1GRN	Kd=388nM	-8.74	2WFX	Kd=14nM	-10.707
5N48	Kd=5nM	-11.317	4Y61	Kd=355.5nM	-8.792
3N4I	Kd=1.1nM	-12.213	2KA6	Kd=52nM	-9.93
5TOJ	Kd=23nM	-10.413	6J56	Kd=0.67uM	-8.417
5LPU	Kd=7.8uM	-6.964	2PON	Kd=1.4uM	-7.981
2VSM	Kd=35nM	-10.165	3GJ8	Kd=3uM	-7.529
3VYR	Kd=140nM	-9.344	2WWK	Kd=1.28uM	-8.034

PDB entry	label	ΔG	PDB entry	label	ΔG
1R6Q	Kd=0.33uM	-8.836	1ZMY	Kd=7.6nM	-11.069
6J9H	Kd=42nM	-10.057	4YL8	Kd=5.4uM	-7.181
5YC5	Kd=180nM	-9.195	1YDI	Kd=1.78nM	-11.928
3E2L	Ki=69pM	-13.853	5XSQ	Kd=7.35nM	-11.089
5HPS	Kd=322.9nM	-8.849	5IJ9	Kd=13.4uM	-6.643
2K7A	Kd=0.67mM	-4.327	4JEH	Kd=8.1nM	-11.031
4DH2	Kd=32.6nM	-10.207	3ECH	Kd=300nM	-8.893
1OYH	Kd=42nM	-10.057	6E2P	Kd=18.7uM	-6.446
5MA5	Kd=29pM	-14.366	3KJ0	Kd=2nM	-11.859
3IM4	Kd=48nM	-9.978	1Y3D	Ki=0.56nM	-12.613
4HCP	Kd=9.4uM	-6.853	1V18	Kd=10nM	-10.906
4GN3	Kd=4.6uM	-7.276	2B42	Kd=1.07nM	-12.23
2QEJ	Kd=1.1nM	-12.213	3CQG	Kd=4nM	-11.449
2F31	Kd=102nM	-9.531	6K4K	Kd=6.51nM	-11.161
4HFK	Kd=0.269nM	-13.047	3PVM	Kd=42nM	-10.057
4NST	IC50=1.37uM	-7.993	5JHW	IC50=0.3nM	-12.983
5XV8	Kd=71.4nM	-9.743	5DFW	Kd=0.5nM	-12.68
1YX5	Kd=350uM	-4.711	3UKZ	Kd=2.4nM	-11.751
3HUG	Kd=20nM	-10.496	5X4L	Kd=2.34uM	-7.676
4K1R	Kd=10.2uM	-6.805			

Supplementary Table 5: The PDB entries, labels, and the corresponce Gabbs free enegy(ΔG) of the PP (protein-protein interaction) dataset used in our study. This dataset was constructed based on protein-protein complexes of PDBbind2020.

PPI family(s)	Uniprot ID
ttr	P02766
hif1a/p300	Q16665/Q09472
wdr5/mll	P61964/Q03164
RUNX1/CBFb	Q01196/Q13951
cd80/cd28	P33681/P10747
ras/sos1	P01112/Q07889
BRI1	Q942F3
VEGF/VEGFR	P15692/P17948
il2/il2r	P60568/P01589
CD40L	trimer : P29965
Tubulin	P02554
PCNA trimer	P12004
cyclophilins	P62937
bromodomain/histone	O60885/P62805
integrins	P56199
SPIN1/Q5TEC6	Q9Y657/Q5TEC6
cd4/gp120	P01730/P04578
bcl2-like/bak	P10415/Q07812
E2/E1	P06790/P06789
MLLT1/H3	Q03111/P68431
annexin/s100	P07355/P60903
SOD1	P00441
brd2/ack	P25440/Q07912
TNF	P01375
WDR5/MLL	P61964/Q03164
xiap/smac	P98170/Q9NR28
menin/mll	O00255/Q03164
Beta-catenin/TCF-4	P35222/P15884
CRM1/Rev	O14980/Q9UBZ9
mdm2-like/p53	Q00987/P04637
NRP/VEGF	Q9QWJ9/P15692
fkbp1a/fk506	P62942/Q02790
lfa/icam	P20701/P05362
stat3	P40763
ledgf/in	O75475/P12497
UPAR/UPA	Q03405/P00749
Myc/Max	P01106/P61244
WD40/H3	O75530/P68431
CaM/CaMBD2	P07463/Q9H2S1
53BP1/H4	Q12888/Q12888
Rac1/GEFs	P63000/Q8SSW7

Supplementary Table 6: Representations of protein families. The selected proteins are provided with Uniprot entries.

References

[1] Fawcett, T. An introduction to roc analysis. *Pattern recognition letters* **27**, 861–874 (2006).

[2] Zhao, N., Zhuo, M., Tian, K. & Gong, X. Protein–protein interaction and non-interaction predictions using gene sequence natural vector. *Communications Biology* **5**, 1–11 (2022).

[3] Guo, Y., Yu, L., Wen, Z. & Li, M. Using support vector machine combined with auto covariance to predict protein–protein interactions from protein sequences. *Nucleic acids research* **36**, 3025–3030 (2008).

[4] Yang, L., Xia, J.-F. & Gui, J. Prediction of protein-protein interactions from protein sequence using local descriptors. *Protein and Peptide Letters* **17**, 1085–1090 (2010).

[5] You, Z.-H., Lei, Y.-K., Zhu, L., Xia, J. & Wang, B. Prediction of protein-protein interactions from amino acid sequences with ensemble extreme learning machines and principal component analysis. In *BMC bioinformatics*, vol. 14, 1–11 (Springer, 2013).

[6] You, Z.-H. *et al.* Prediction of protein-protein interactions from amino acid sequences using a novel multi-scale continuous and discontinuous feature set. In *BMC bioinformatics*, vol. 15, 1–9 (Springer, 2014).

[7] Wong, L., You, Z.-H., Li, S., Huang, Y.-A. & Liu, G. Detection of protein-protein interactions from amino acid sequences using a rotation forest model with a novel pr-lpq descriptor. In *International Conference on Intelligent Computing*, 713–720 (Springer, 2015).

[8] Du, X. *et al.* Deepppi: boosting prediction of protein–protein interactions with deep neural networks. *Journal of chemical information and modeling* **57**, 1499–1510 (2017).

[9] Wang, Y. *et al.* Pcvmzm: using the probabilistic classification vector machines model combined with a zernike moments descriptor to predict protein–protein interactions from protein sequences. *International Journal of Molecular Sciences* **18**, 1029 (2017).

[10] Hashemifar, S., Neyshabur, B., Khan, A. A. & Xu, J. Predicting protein–protein interactions through sequence-based deep learning. *Bioinformatics* **34**, i802–i810 (2018).

[11] Chen, C., Zhang, Q., Ma, Q. & Yu, B. Lightgbm-ppi: Predicting protein-protein interactions through lightgbm with multi-information fusion. *Chemometrics and Intelligent Laboratory Systems* **191**, 54–64 (2019).

[12] Chen, M. *et al.* Multifaceted protein–protein interaction prediction based on siamese residual rcnn. *Bioinformatics* **35**, i305–i314 (2019).

[13] Chen, C. *et al.* Improving protein-protein interactions prediction accuracy using xgboost feature selection and stacked ensemble classifier. *Computers in Biology and Medicine* **123**, 103899 (2020).

[14] Song, B. *et al.* Learning spatial structures of proteins improves protein–protein interaction prediction. *Briefings in Bioinformatics* (2022).

[15] Nanni, L. Hyperplanes for predicting protein–protein interactions. *Neurocomputing* **69**, 257–263 (2005).

[16] Huang, Y.-A., You, Z.-H., Gao, X., Wong, L. & Wang, L. Using weighted sparse representation model combined with discrete cosine transformation to predict protein-protein interactions from protein sequence. *BioMed research international* **2015** (2015).

[17] Martin, S., Roe, D. & Faulon, J.-L. Predicting protein–protein interactions using signature products. *Bioinformatics* **21**, 218–226 (2005).

[18] Bock, J. R. & Gough, D. A. Whole-proteome interaction mining. *Bioinformatics* **19**, 125–134 (2003).

[19] Zhao, X., Tian, K., He, R. L. & Yau, S. S.-T. Establishing the phylogeny of prochlorococcus with a new alignment-free method. *Ecology and evolution* **7**, 11057–11065 (2017).

[20] Najafabadi, H. S. & Salavati, R. Sequence-based prediction of protein-protein interactions by means of codon usage. *Genome biology* **9**, 1–9 (2008).

[21] Berman, H. M. *et al.* The protein data bank. *Nucleic acids research* **28**, 235–242 (2000).

[22] Rodrigues, C. H., Pires, D. E. & Ascher, D. B. pdcsm-ppi: Using graph-based signatures to identify protein–protein interaction inhibitors. *Journal of Chemical Information and Modeling* **61**, 5438–5445 (2021).
