# Supplementary material for: SVSBI: sequence-based virtual screening of biomolecular interactions
Source: Commun Biol. 2023 May 18;6:536. doi: 10.1038/s42003-023-04866-3 (PMC10195826; doi:10.1038/s42003-023-04866-3)
Supplement: Supplementary file 5 — Reporting Summary [file 42003_2023_4866_MOESM5_ESM.pdf]

nature portfolio

Corresponding author(s): Guo-Wei Wei

Last updated by author(s): Feb 28, 2023

Reporting Summary

Nature Portfolio wishes to improve the reproducibility of the work that we publish. This form provides structure for consistency and transparency in reporting. For further information on Nature Portfolio policies, see our Editorial Policies and the Editorial Policy Checklist.

Statistics

For all statistical analyses, confirm that the following items are present in the figure legend, table legend, main text, or Methods section.

n/a	Confirmed	
☐	☒	The exact sample size (*n*) for each experimental group/condition, given as a discrete number and unit of measurement
☐	☒	A statement on whether measurements were taken from distinct samples or whether the same sample was measured repeatedly
☒	☐	The statistical test(s) used AND whether they are one- or two-sided<br>*Only common tests should be described solely by name; describe more complex techniques in the Methods section.*
☒	☐	A description of all covariates tested
☐	☒	A description of any assumptions or corrections, such as tests of normality and adjustment for multiple comparisons
☐	☒	A full description of the statistical parameters including central tendency (e.g. means) or other basic estimates (e.g. regression coefficient) AND variation (e.g. standard deviation) or associated estimates of uncertainty (e.g. confidence intervals)
☒	☐	For null hypothesis testing, the test statistic (e.g. *F*, *t*, *r*) with confidence intervals, effect sizes, degrees of freedom and *P* value noted<br>*Give P values as exact values whenever suitable.*
☒	☐	For Bayesian analysis, information on the choice of priors and Markov chain Monte Carlo settings
☒	☐	For hierarchical and complex designs, identification of the appropriate level for tests and full reporting of outcomes
☐	☒	Estimates of effect sizes (e.g. Cohen's *d*, Pearson's *r*), indicating how they were calculated

Our web collection on statistics for biologists contains articles on many of the points above.

Software and code

Policy information about availability of computer code

Data collection	HS, MM, SC, DM, HP collections are from NVDT server: https://github.com/Zhaonan99/NVDT.<br>PP, PL, PN collections are from the PDBBind database server: http://www.pdbbind.org.cn/.<br>iPPI collection is from pdCSM-PPI server: https://biosig.lab.uq.edu.au/pdcsm_ppi/.
Data analysis	Open source code used in this study: pytorch(1.10.0),scikit-learn(1.1.1),scipy(1.8.1),numpy(1.22.3) ,pandas(1.5.0), esm(0.3.1), tensorflow(1.13.0&2.2.0), dnabert(https://github.com/jerryji1993/DNABERT), PretrainModels(https://github.com/WeilabMSU/PretrainModels), antoencoder-v01(https://github.com/WeilabMSU/antoencoder-v01).<br>Source code of this study is available at http://github.com/WeilabMSU/SVS.

For manuscripts utilizing custom algorithms or software that are central to the research but not yet described in published literature, software must be made available to editors and reviewers. We strongly encourage code deposition in a community repository (e.g. GitHub). See the Nature Portfolio guidelines for submitting code & software for further information.

Data

Policy information about availability of data

All manuscripts must include a data availability statement. This statement should provide the following information, where applicable:

- Accession codes, unique identifiers, or web links for publicly available datasets
- A description of any restrictions on data availability
- For clinical datasets or third party data, please ensure that the statement adheres to our policy

All datasets are available at https://weilab.math.msu.edu/DataLibrary/2D/.

Human research participants

Policy information about studies involving human research participants and Sex and Gender in Research.

Reporting on sex and gender	N/A
Population characteristics	N/A
Recruitment	N/A
Ethics oversight	N/A

Note that full information on the approval of the study protocol must also be provided in the manuscript.

Field-specific reporting

Please select the one below that is the best fit for your research. If you are not sure, read the appropriate sections before making your selection.

☒ Life sciences ☐ Behavioural & social sciences ☐ Ecological, evolutionary & environmental sciences

For a reference copy of the document with all sections, see nature.com/documents/nr-reporting-summary-flat.pdf

Life sciences study design

All studies must disclose on these points even when the disclosure is negative.

Sample size	The size of data is based on the available data from literature cited.
Data exclusions	The PN dataset is generated from PDBbind2020 based on the following criterion: 1) exclude those protein-nucleic acid complexes that their sequence numbers do not equal two; 2) exclude those protein-nucleic acid complexes that have unclear labels; 3) exclude those protein-nucleic acid complexes that have abnormal letters (normal ones are A, C, T, G) in its nucleic-acid sequences; 4) exclude those protein-nucleic complexes that whose nucleic acid sequence length is fewer than 6.
Replication	Al replication of our works follow previous standard procedures and details can be found in the Supplement.
Randomization	All cross-validations are done by sci-learn with random seeds.
Blinding	We are blinded to group allocation during data collection and analysis.

Reporting for specific materials, systems and methods

We require information from authors about some types of materials, experimental systems and methods used in many studies. Here, indicate whether each material, system or method listed is relevant to your study. If you are not sure if a list item applies to your research, read the appropriate section before selecting a response.

Materials & experimental systems

n/a	Involved in the study	
☒	☐	Antibodies
☒	☐	Eukaryotic cell lines
☒	☐	Palaeontology and archaeology
☒	☐	Animals and other organisms
☒	☐	Clinical data
☒	☐	Dual use research of concern

Methods

n/a	Involved in the study	
☒	☐	ChIP-seq
☒	☐	Flow cytometry
☒	☐	MRI-based neuroimaging
